# Fluconazole in hypercalciuric patients with increased 1,25(OH)_2_D levels: the prospective, randomized, placebo-controlled, double-blind FLUCOLITH trial

**DOI:** 10.1186/s13063-022-06302-z

**Published:** 2022-06-16

**Authors:** Aurélia Bertholet-Thomas, Aurélie Portefaix, Sacha Flammier, Carole Dhelens, Fabien Subtil, Laurence Dubourg, Valérie Laudy, Myrtille Le Bouar, Inesse Boussaha, Marietou Ndiaye, Arnaud Molin, Sandrine Lemoine, Justine Bacchetta

**Affiliations:** 1 Centre de Référence des Maladies Rénales Rares, filières maladies rares ORKID and ERK-Net, Service de Néphrologie, Rhumatologie et Dermatologie Pédiatriques, Hôpital Femme Mère Enfant, Boulevard Pinel, 69677 Bron, Cedex France; 2 Centre de Référence des Maladies Rares du Calcium et du Phosphate, filière maladies rares OSCAR, Hôpital Femme Mère Enfant, Bron, France; 3 INSERM 1033, Prévention des Maladies Osseuses, Lyon, France; 4 Centre d’Investigation Clinique, Hôpital Cardiovasculaire Louis Pradel, Inserm 1407, 69500 Bron, France; 5 Pharmacie, FRIPHARM, Hôpital Edouard Herriot, Lyon, France; 6 Service de Biostatistique, Hospices Civils de Lyon, Lyon, France; 7 Université de Lyon, Université Lyon 1, CNRS, Laboratoire de Biométrie et Biologie Évolutive UMR 5558, Villeurbanne, France; 8 Néphrologie, Dialyse, Hypertension Artérielle et Exploration Fonctionnelle Rénale, Hôpital Edouard Herriot, Lyon, France; 9 Faculté de Médecine Lyon Est, Université Lyon 1, Lyon, France; 10 Service de Génétique, CHU de Caen, Caen, France; 11 Centre de Référence des Maladies Rares du Calcium et du Phosphate, filière maladies rares OSCAR Caen, Caen, France

**Keywords:** SLC34A1, SLC34A3, CYP24A1, Hypercalciuria, Fluconazole, 1,25(OH)_2_D, Phosphate, Randomized, Controlled, Nephrolithiasis

## Abstract

**Background:**

Hypercalciuria is one of the most frequent metabolic disorders associated with nephrolithiasis and/or nephrocalcinosis possibly leading to chronic kidney disease (CKD) and bone complications in adults. Orphan diseases with different underlying primary pathophysiology share inappropriately increased 1,25(OH)_2_D levels and hypercalciuria, e.g., hypersensitivity to vitamin D and renal phosphate wasting. Their management is challenging, typically based on hyperhydration and dietary advice.

The antifungal azoles are known to inhibit the 1α-hydroxylase and therefore decrease 1,25(OH)_2_D levels; they are commonly used, with well described pharmacokinetic and tolerability data. Fluconazole has been successfully reported to reduce calciuria in patients with *CYP24A1* or *SLC34A3* mutations, with no safety warnings. Thus, based on these case reports, we hypothesize that fluconazole is effective to decrease and normalize calciuria in patients with hypercalciuria and increased 1,25(OH)_2_D levels.

**Methods:**

The FLUCOLITH trial is a prospective, interventional, randomized in parallel groups (1:1), placebo-controlled, double-blind trial. A total of 60 patients (10–60 years) with nephrolithiasis and/or nephrocalcinosis history, hypercalciuria (> 0.1 mmol/kg/day), increased 1,25(OH)_2_D levels (> 150 pmol/L), and 25-OH-D levels >20 nmol/L will be included. Inclusions will be performed only from mid-September to the beginning of February to avoid bias due to sunlight-induced vitamin D synthesis. The primary endpoint will be the proportion of patients with normalization of 24-h calciuria between baseline and 16 weeks, or with a relative decrease of at least 30% of 24-h calciuria in patients who still display at W16 a 24-h hypercalciuria.

**Discussion:**

The current challenge is to propose an efficient treatment to patients with hypercalciuria and increased 1,25(OH)_2_D levels in order to prevent later complications and notably CKD that can ultimately lead to end-stage renal disease. Based on improvement of knowledge in phosphate/calcium metabolism, pathophysiology and genetics, the “off-label” use of fluconazole was recently reported to be useful in hypercalciuric patients with increased 1,25(OH)_2_D levels. Thus, the FLUCOLITH study is a unique opportunity to develop a new indication of a well-known and not expensive drug in orphan renal diseases, the ultimate objective being the secondary prevention of CKD worsening in these patients.

**Trial registration:**

ClinicalTrials.gov NCT04495608. Registered on July 23, 2020.

**Supplementary Information:**

The online version contains supplementary material available at 10.1186/s13063-022-06302-z.

## Background

Hypercalciuria is one of the most frequent metabolic disorders associated with nephrolithiasis and/or nephrocalcinosis possibly leading to chronic kidney disease (CKD) and bone complications in adults. Due to the availability of genetic testing and improvement of genetic knowledge, some “idiopathic” cases with a mild phenotype are now explained by mutations in *CYP24A1* (encoding the vitamin D 24-hydroxylase enzyme) or *SLC34A1/SLC34A3* encoding the sodium-phosphate cotransporters Npt2a and Npt2c, respectively, and responsible for phosphate reabsorption in renal proximal tubular cells [1, 2].

Hypercalciuria can be secondary to increased intestinal absorption and/or increased renal distal tubular reabsorption of calcium due to increased active vitamin D, i.e., 1,25(OH)_2_D, levels. The putative mechanisms responsible for high 1,25(OH)_2_D levels in kidney stone formers and nephrocalcinosis have been recently reviewed [3]. Briefly, in physiology, fibroblast growth factor (FGF23) inhibits 1,25(OH)_2_D through at least two direct mechanisms: an inhibition of the 1-α hydroxylase and a stimulation of the 24-hydroxylase, thus leading to decreased 1,25(OH)_2_D levels. In contrast, 1,25(OH)_2_D is stimulated by (absolute or relative) hypocalcemia, (absolute or relative) hypophosphatemia, and parathyroid hormone (PTH). Even though they have a different pathophysiology, some diseases share inappropriate increased 1,25(OH)_2_D levels as a direct or indirect result of the underlying defect [3]. This is the case for example for hypersensitivity to vitamin D (*CYP24A1* mutations, or neonatal severe hypercalcemia without genetic explanation), hypercalciuria with renal phosphate wasting (mutations in the genes encoding Npt2a, Npt2c, and NHERF1), or the exceptional familial tumoral calcinosis (low levels of active or resistance to FGF23) [4]. In patients with mutations in the genes encoding Npt2a or Npt2c, the decreased tubular phosphate reabsorption induces both a “downregulation” of FGF23 (which in turn decreases the inhibition of 1,25(OH)_2_D by FGF23) and a direct stimulation of 1,25(OH)_2_D to increase intestinal phosphate absorption to maintain circulating phosphate levels, even in the absence of overt hypophosphatemia. Thus, these two pathways explain the increased 1,25(OH)_2_D levels observed in these patients. Longitudinal data of patients with *CYP24A1* mutations have also suggested that, in most patients, periods of increased sunlight exposure tended to correlate with decreases in PTH levels and increases in both circulating and urinary calcium [5].

The management of these genetic causes of hypercalciuria usually rely on hyperhydration and dietary advice, notably low-sodium diet with normal calcium intake for age. In case of overt hypophosphatemia and subsequent abnormal bone mineralization, phosphate supplementation may be given to these patients, at least during childhood, similarly to what is done in patients with hypophosphatemic rickets [6]. Other strategies such as hydrochlorothiazide can be proposed, however with an uncertain medical benefit in view of side effects (hypokalemia, asthenia, potential cutaneous long-term side effects) [7].

Azoles are known to inhibit the 1α-hydroxylase and therefore decrease 1,25(OH)_2_D levels. These antifungal drugs are commonly used in neonates, infants, and adults; pharmacokinetic data are well described. In the early 2010s, ketoconazole was reported to decrease calciuria in patients with vitamin D hypersensitivity [8, 9]. However, its off-label use may be challenging, mainly because of its potential liver toxicity on the long term [10]. More recently, to improve azole tolerance, fluconazole has been successfully reported to reduce urinary calcium in a few patients with *CYP24A1* or *SLC34A3* mutations, while maintaining a stable renal function [3, 11].

Based on these observations, we hypothesize that fluconazole is effective to decrease and normalize calciuria in patients with hypercalciuria and increased 1,25(OH)_2_D levels. We will therefore conduct a placebo-controlled double-blind trial testing fluconazole in hypercalciuric patients with increased 1,25(OH)_2_D levels.

## Methods/design

**Fig. 1:**
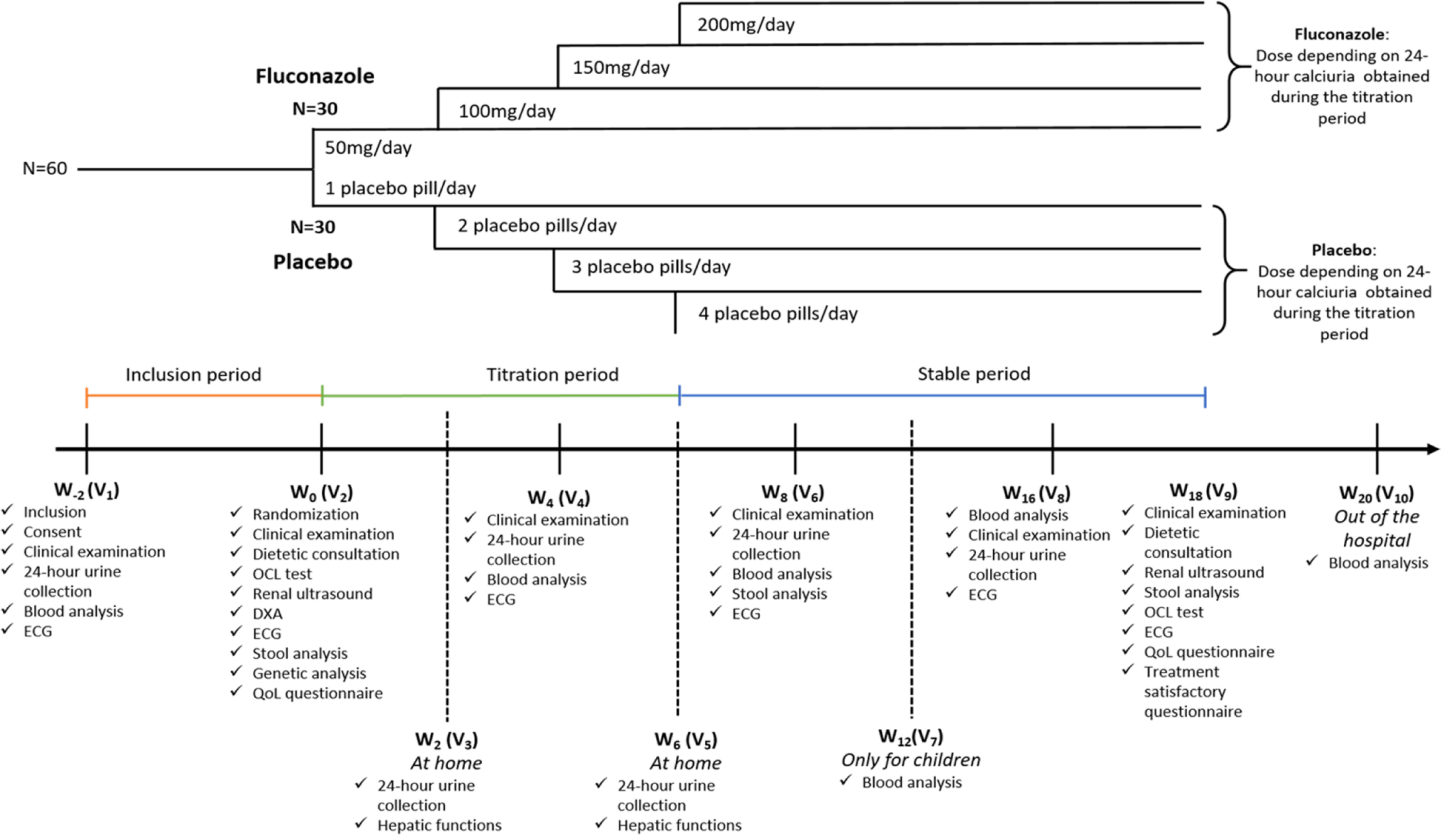
Overall summary of the FLUCOLITH trial

The FLUCOLITH trial is a phase 2, prospective, interventional, national, randomized in parallel groups (1:1), placebo-controlled, exploratory double-blind trial, as summarized in Fig. 1.

The national French recruitment will be ensured through two national rare disease networks (OSCAR and ORKiD networks); the participation of 26 French University Hospitals is expected, with physicians from Nephrology and Endocrinology Departments, and from adult and pediatric units (cf. Additional file 2 - List of study sites). The trial is supported by a grant from the French Ministry of Health (*Programme Hospitalier de Recherche Clinique National* 2019) and the study sponsor is the Hospices Civils de Lyon (Lyon, France). The funding body and the study sponsor are not involved in the design of the study and collection, analysis and interpretation of data, and the writing the protocol.

### Participants

#### Eligibility criteria

The inclusion criteria will be the following: patients who presented in their medical history nephrolithiasis and/or nephrocalcinosis; patients who had, at least 4 weeks (±2 weeks) before inclusion and at inclusion (V_1_), a local biological evaluation with 24-h urine calcium above 0.1 mmol/kg/day, and 1,25(OH)_2_D levels above or equal to 150 pmol/L, and 25-OH-D levels above or equal to 20 nmol/L, and calcemia levels below or equal to 2.65 mmol/L; children from 10 years or adults until 60 years; women using effective methods of contraception during the study period, partners of male patients of child-bearing potential must use highly effective methods of contraception; patients insured or beneficiary of a health insurance plan; evidence of signed and dated informed consent document(s) indicating that the subject and/or his parents/legal guardian have/has been informed of all pertinent aspects of the trial.

The non-inclusion criteria will be the following: patient who already received fluconazole or ketoconazole during the last 6 months before inclusion; patients weight below 28 kg; patients with BMI>35; patients who cannot stop hydrochlorothiazide or other diuretics during the screening and study period; patients who cannot stop vitamin D supplementation and/or calcium supplementation (drugs, enriched waters, etc.) during the study period; hypersensitivity to fluconazole and/or other derivative azoles and/or excipients; due to the presence of lactose excipient, patients presenting rare hereditary abnormalities of galactose intolerance, of Lapp lactase deficit, or of glucose-galactose malabsorption; patients who need co-administration with other drugs known to prolong the QT interval and metabolized by cytochrome P450 (CYP3A4) such as pimozide, quinidine, and erythromycin; patients with iatrogenic hypercalciuria (vitamin D intoxication, immobilization); patients with risk of QT interval prolongation (congenital Long QT syndrome, familial history of sudden cardiac death before 50 years of age, cardiopathy: ischemia or myocardial infarction, congestive cardiac insufficiency, left ventricle hypertrophy, cardiomyopathy, conduction trouble within 6 months preceding the inclusion, arrhythmia history, electrolytic instabilities; bradycardia (< 50 beats per minute), acute neurological events within 6 months preceding the inclusion, adult patients with a QT interval/corrected QT interval > 470 ms for women and > 450 ms for men at the ECG performed at the inclusion visit (V_1_), or children from 10 years with a QT interval/corrected QT interval should be > 460 ms for girls and > 450 ms for boys); children with a history of cardiac pathology, patients with an estimated glomerular filtration rate < 60 mL/min/1.73 m^2^; patients with a liver disease or an abnormality in the initial liver lab test; patients with enuresis; patients with another cause of identified nephrolithiasis; patients suffering from granulomatosis pathology such as sarcoidosis; patients with hyperparathyroidism; women who are pregnant or breast feeding, or who have a project of pregnancy before the end of the study; women menopaused; patients with a project of travelling in a sunny area during the study period; immunodeficient patients, patients with other diseases or disorders that could preclude assessment; patients who are participating in another research study that may interfere with the results or conclusions of this study; and patients under judicial protection.

### Intervention

The patients likely to participate in the study will be identified within the Nephrology and the Endocrinology Departments of the participating CHU, as part of their regular medical follow-up. Inclusions will be performed only from mid-September to the beginning of February so as to avoid bias due to sunlight-induced vitamin D synthesis.

Patients will receive fluconazole or placebo during 18 weeks.

A therapeutic adjustment will be done during the first 6 weeks of treatment (titration period) according to the 24-h calciuria results. Treatment will be modified if necessary by increasing the number of capsules. Treatment will start with 50 mg/day until maximum 200 mg/day. Patients allocated will receive between 1 and 4 capsules of fluconazole/placebo during the first 6 weeks of the trial.

The site personnel will directly enter calciuria results onto the eCRF. During the titration period (week 2 (V_3_), week 4 (V_4_), and week 6 (V_5_)), the posology to adopt will be automatically given by the eCRF, via an algorithm common to both treatment groups:

- If 24-h calciuria is > 0.1mmol/kg/day, fluconazole/placebo dose will be increased to 50 mg per day until the next visit,
- If 24-h calciuria is ≤ 0.1mmol/kg/day, fluconazole/placebo dose will remain stable until the next visit.

From week 6 (V_5_) and until the end of the treatment period (week 18, V_9_), the treatment dose will remain stable (stable period), as illustrated in Fig. 1.

Temporary or permanent discontinuation of investigational medical product can occur due to biological lab abnormalities (calcemia levels > 2.65 mmol/L after two tests, hepatic toxicity, neutrophil counts < 1000 cells/μL, platelet count < 150,000 cells/μL), all anaphylactic reaction, cardiac abnormalities observed on the ECG exam, renal insufficiency with creatinine clearance <50 mL/min/1.73 m^2^, significant vitamin D level reduction (25-OH-D level <20nmol/L), or hypophosphatemia.

Blinding procedure will be systematic thanks to the indistinguishable nature of the active product and placebo and their packaging. Participant, care provider, investigator, outcomes assessor, and the coordinating center are blinded to the arm of treatment. Since patients (and their representatives if applicable) and investigators are blinded to the treatment arm, this is referred to as a double-blind study even if all other participants are also blinded. Only the biostatistician in charge of the production of the randomization list, the Centre Anti-Poison of Lyon, and the main pharmacy (Pharmacy Department Groupement Hospitalier Centre – Edouard Herriot Hospital – Hospices Civils de Lyon (Lyon, France), responsible for packaging, labelling, and dispatching of experimental drugs to the sites will have access to a decoded list. Unblinding will be possible 24 h/7 days simply by phone call to the Centre Anti-Poison de Lyon (CAP - 04 72 11 69 11). The CAP physician will be able to proceed to the unblinding if required.

The examinations realized during the visits will be performed specifically for the study: urine, and blood analysis, electrocardiogram, dual X-ray absorptiometry (DXA), renal ultrasounds, dietetic questionnaires, oral calcium load tests [12]. Bone evaluation will be done with DXA: femoral neck (FN), lumbar spine vertebra 2 to 4 (LS2-4), and total body (TB) areal bone mineral density (aBMD, g cm^−2^). For *Z*-scores for TB and LS2-4, aBMD will be calculated depending of age and gender. DXA will be performed locally and we will only keep the results as Z-scores from the different centers, provided that DXA scans are locally performed according to the ISCD (International Society of Clinical Densitometry) guidelines. All DXA images will be anonymized by the investigator sites and centralized on an internet platform. The investigators will only have an access to their patients. A central review of all DXA results will be done by an engineer from INSERM Unit 1033 “pathophysiology, diagnosis and treatment of musculoskeletal disorders” in Edouard Herriot Hospital in Lyon.

The investigational medicinal product (IMP) is fluconazole. For the study, the IMP and its placebo will be provided in 22 capsule numbered bottles. Placebo will be used as treatment of comparison as there is no gold standard strategy.

Fluconazole 50 mg and placebo capsules are provided, in the same appearance (color, size, and packaging) by the sponsor. The IMP and placebo are prepared, blinded, labelled, and delivered in every participating center by the Pharmacy of the Edouard Herriot Hospital (FRIPHARM, Hospices Civils Lyon, France), authorized for the compounding of experimental drugs. Preparation of the IMP is realized according to good preparation practices from tablets of Fluconazole ARROW® 50 mg, supplied by ARROW (France). The placebo capsules are composed of lactose (excipients in the labelling and package leaflet of medicinal products for human use).

Co-administration with medicinal products known to prolong the QT interval and metabolized by cytochrome P450 (CYP) 3A4 is contraindicated in patients treated with fluconazole. Halofantrine, Amiodarone, and medications used to treat the pathology are not recommended too.

### Objectives and endpoints

The primary objective is to demonstrate that fluconazole normalizes or decreases urinary calcium after 4 months of treatment in patients with hypercalciuria and increased 1,25(OH)_2_D levels. The primary endpoint will be the proportion of patients with normalization of 24-h urinary calcium between baseline and 16 weeks (W_16_), or with a relative decrease of 30% of 24-h urinary calcium in patients who still display hypercalciuria (> 0.1 mmol/kg/day) at W_16_.

The secondary objectives are to evaluate the effects of fluconazole on the evolution of biomarkers of calcium/phosphate metabolism and renal function over time, to describe precisely the cohort at baseline and after 4 months of treatment, to assess safety of fluconazole, to identify the onset of potential mycological resistances, to assess compliance of treated patients, and to measure quality of life and treatment satisfaction. In order to answer to the secondary objectives, corresponding secondary endpoints (as summarized in Table 1) will be collected.

**Table 1:** Secondary objectives and endpoints of the FLUCOLITH trial

Secondary objectives	Secondary endpoints to collect
1- Effects of fluconazole on the evolution of calcium/phosphate metabolism over time	- Serum analysis: calcium, ionized calcium, phosphate, magnesium, PTH, 25-OH-D, 1,25(OH)_2_D, 24-25(OH)_2_D, 25-OH-D:24-25(OH)_2_D ratio, total alkaline phosphatase - 24-h urine collection: phosphate, calcium, creatinine, TmP/GFR, citrate - OCL test
2- Evolution of renal function	- Serum creatinine allowing the calculation of eGFR with the FAS formula - Renal ultrasounds: number and size of lithiasis, nephrocalcinosis
3- Detailed description of the cohort at baseline and after treatment	- Anthropometry - Evaluation of nutritional intakes: calcium, sodium and protein intakes estimated with a dietetic evaluation and completion of 3 questionnaires - Bone evaluation with biomarkers: bone alkaline phosphatases, FGF23, Klotho - Bone evaluation with DXA - Genetic analysis (if not already performed)
4- Safety evaluation	- Cardiac evaluation: electrocardiogram, corrected QT interval - Monthly blood analyses: hepatic functions, complete blood cell counts, albumin, serum creatinine, calcium, phosphate, LDH
5- Evaluation of the onset of potential mycological resistances	Mycological samples (urine and buccal samples) to evaluate the onset of potential resistance of candida
6- Compliance assessment	- Accountability of returned study treatment - Information of patients’ diary
7- Quality of life and treatment satisfaction assessments	- Quality of life questionnaires (8–12 years, 13–17 years, ≥ 18 years) reported by patients - Treatment satisfactory questionnaire reported by patients

*PTH* parathyroid hormone*, 25-OH-D* 25 OH vitamin D, *1,25(OH)*_*2*_*D* 1-25 di-hydroxy-vitamin D, *24-25(OH)*_*2*_*D* 24-25 di-hydroxy-vitamin D, *OCL* oral calcium load, *TmP/GFR* Tubular maximum Phosphate Reabsorption per Glomerular Filtration Rate, *eGFR* estimated glomerular filtration rate, *FGF23* Fibroblast Growth Factor 23, *DXA* dual X-ray absorptiometry, *LDH* lactate dehydrogenase

### Benefit/risk ratio

The benefit for the patients is a standardized and complete medical follow-up and monitoring during the study period. Moreover, if fluconazole is effective, recovering normal urinary calcium levels will decrease the risk of further nephrolithiasis and/or nephrocalcinosis, CKD, and bone complications. There is no major risk expected for the participants. The study includes non-invasive and low-radiation radiological examinations (DXA and renal ultrasounds).

The patients will undergo additional blood and urine analyses as compared to usual standard practice. The blood volume taken for the study will not exceed the threshold volume defined by the Blood Volume Guidelines. Fluconazole is supposed to decrease urinary calcium by decreasing 1,25(OH)_2_D concentrations; however, in this population with increased 1,25(OH)_2_D levels at baseline, the risk of 1,25(OH)_2_D deficiency induced by fluconazole treatment appears very low. To monitor the possible emergence of *Candida* resistance, urine and buccal samples will be collected before the treatment period (W_0_) and after 8 and 16 weeks of treatment. Thus, the benefit/risk ratio does not seem to be unfavorable.

### Randomization

The randomization will be stratified on the age group (children [10;18], adult [18;60]). Randomization will be performed centrally through the Ennov Clinical software. The lists for randomization will be provided by the department of biostatistics of the Hospices Civils de Lyon and built using the permuted block randomization method. The data from the 5 first treated adults during a period of 10 weeks after titration period (visit V_8_) will be at first evaluated by the DSMB and reported to ANSM to decide inclusion of patients < 18 years old.

### Sample size

A total of 60 patients (adults and children) will be included.

The sample size calculation was performed according to these hypotheses: 15% of normalization of calciuria (≤0.1 mmol/kg/day) or decrease of 30% of calciuria (as compared to baseline) is expected in the placebo group vs 60% in the fluconazole group. Under these hypotheses, 25 patients should be randomized per group to achieve 89% power to show a statistical difference between the two groups (two-sided 5% alpha level, Fisher test).

Amoung the patients included at V_1_, only patients with 24-h calciuria > 0.1 mmol/kg/day and serum 1,25(OH)_2_D levels ≥ 150 pmol/L and serum 25-OH-D levels > 20 nmol/L and calcemia levels ≤ 2.65 mmol/L will be randomized. Hence, it is planned to recruit at V1, 60 patients to be sure to have at least 50 patients randomized. Moreover, it will allow to take into account lost to follow-up in the study.

### Data management

There is a priori no criterion for premature drop-out. The patients, regardless of the randomization group, will not be excluded from the study and will be followed up according to the protocol.

Outcomes related to 24-h calciuria measurements will be collected and reported at baseline and before every study visits V_3_, V_4_, V_5_, V_6_, and V_8_, by the investigator or the authorized persons in the medical field of participant, as per site practice using a standard of care software. If there is missing data due to a problem during the collection of urine, a 4-day margin is planned to be able to organize a new collection at the patient’s home/center.

All study results collected in electronic or paper source documents will be entered in an electronic case report form by the investigator or an authorized person who will be appearing on the tasks delegation sheet, as soon as they are collected (during/after patients’ visits). Data will be coded with respect to data confidentiality. Each form will be dated and signed electronically by the investigator, signifying its agreement with the data entered in the eCRF. The Ennov Clinical system was selected. Control quality of the data is performed both centrally and on site by monitors. Presence, accuracy, and conformity of the data is verified.

Surveillance and emergence of adverse and serious adverse event is performed. All adverse and serious adverse events will be reported in the eCRF. The investigator evaluates each adverse event in terms of severity, causality with experimental drug (reasonably related / unrelated). All the serious adverse events shall be notified without delay to the sponsor.

The sponsor may temporally or definitively interrupt a subject’s participation in the event of a serious adverse event.

The patients will be discontinued from the study treatment due to calcemia levels, hepatic toxicity, low neutrophil or platelet count, any anaphylactic reaction, cardiac abnormalities, or renal insufficiency.

### Statistical analyses

The intent to treat (ITT) population is defined as all the randomized patients according to their arm randomly allocated, whatever their inclusion criteria, their actual arm, would they be evaluable or not for the primary endpoint. Description of patients at baseline (inclusion) will be done in intent to treat. Primary and secondary endpoints regarding the efficacy of fluconazole will be analyzed in intent to treat. Per-protocol (PP) population is defined as the ITT population without the patients with major protocol deviations. Major protocol deviations will be identified during the blind review and will include, but are not limited to, cases of premature withdrawals and absence of corresponding data, non-respect of eligibility and/or non-eligibility criteria, very poor adherence to study treatment/ study treatment not taken, a late inclusion date leading to the ending of the study during summer, etc. They will be identified during the blind review and will be specified in the statistical analysis plan. Per-protocol analyses will be performed as secondary analysis of the primary endpoint. The safety population is defined as the population of patients with at least one dose of study treatment, analyzed in the actual treatment arm. The safety endpoints will be analyzed on the safety population.

The quantitative variables will be described by the following parameters: number of patients, number of missing values, mean, standard deviation (SD), median, first and third quartiles (Q1 and Q3), minimum, and maximum. The qualitative variables will be described by the following parameters: number of patients, number of missing values, frequency, and percentage of each modality (missing values will not be included in the denominator used for frequency computation). A *p*-value less than 5% will be considered as statistically significant. Two-sided 95% confidence intervals will be provided for the analyses. The analyses will be performed using the R and SAS software.

For the primary outcome, the proportions of patients with normalization of calciuria or decrease of 30% of calciuria (as compared to baseline, V_1_ from W_16_, V_8_) will be described in both groups. The effect of fluconazole on calciuria will be quantified and tested through the odds ratio (with its 95% confidence interval), adjusted on the age class (children/adult) and on the level of 1,25(OH)_2_D during the screening period (150–200 pmol/L or >200 pmol/L). A description of the proportions of success depending on the dose of fluconazole administered at the end of the titration period will be performed. An exploratory analysis of the fluconazole effect will be performed in the subgroups defined by the level of 1,25(OH)_2_D during the screening period (150–200 pmol/L or >200 pmol/L). Secondary endpoints related to evolution over time will be analyzed using mixed effect models.

Missing data will generally not be imputed. If there is missing data due to a problem during the collection of urine (carrier, broken tube...) a 4-day margin is planned to be able to organize a new collection at the patient’s home/center. For the primary endpoint, a patient without calciuria assessment at W_16_ will be considered as a patient without normalization of calciuria. Two sensitivity analyses will be performed. The first one will be performed by multiple imputations, the outcome of patients without 24-h calciuria assessment at W_16_ will be sampled from a Bernoulli distribution with probability equals to the proportion of calciuria normalization in the same treatment, initial 1,25(OH)_2_D level category, and age class groups. A second analysis will be performed by excluding all patients without calciuria assessment at W_16_.

A detailed statistical analysis plan will be written before the database is frozen. It will take into account all protocol modifications or all unexpected events occurring throughout the study and having an impact on the analyses presented here. The planned analyses may be completed in line with the study objectives. They will be carried on by the Biostatistic Department of the Hospices Civils de Lyon.

### Quality control

A Clinical Research Associate mandated by the sponsor will ensure the proper conduct of the study, collection of written data, their documentation, recording, and reporting, in conformity with the Good Clinical Practices as well as the current legal and regulatory provisions.

Furthermore, the investigators agree to accept the quality control audits carried out by persons mandated by the sponsor as well as inspections by the competent authorities. All data and all documents and reports may be the subject of regulatory audits and inspections without the possibility of using medical secrecy as opposition.

### Data safety monitoring board

An independent monitoring committee (DSMB) will be set up. The DSMB will authorize in association with the ANSM the enrollment of pediatric patients in the study (patients <18 years old), once inclusion of 5 adult patients treated during a minimum period of 10 weeks at stable dose (W_16_), in accordance with safety data collected in the study. The DSMB will evaluate the onset of potential mycological resistance among the study and monitor the unexpected adverse events.

It will be made of two specialists of nephrolithiasis, a pharmacologist, and an infectious disease specialist.

Further details can be found in the DSMB Charter (Additional file 3).

### Ethics

The study protocol was first approved by the Ethic committee EC Nord Ouest I on December 14th 2020 (20.09.22.57341), and then on June 17th 2021 (20.00146.057341-MS01) after substantial modifications and by the French competent authority (Agence Nationale de Sécurité du Médicament et des produits de santé, ANSM) on November 24th, 2020 (MEDAECPP-2020-09-00014) and then on June 30th 2021 (MEDMSANAT-2021-06-0053_2020-003011-97). This approval applies for all participating centers. It is registered under EudraCT number 2020-003011-97. The study is compliant with the reference methodology of Commission Nationale de l’Informatique et des Libertés. The study is conducted in accordance with the French legislation, the Good Clinical Practice, and the Declaration of Helsinki. Written consent will be obtained for all patients before inclusion, for their participation to the study research and for the constitution and the storage of a biocollection. For patients who have not yet had a genetic analysis, a specific genetic consent will be proposed, to perform genetic testing and to store corresponding samples, according to current practice.

For minor, consent of both parents (or legal guardian) is required.

The sponsor has subscribed to an insurance policy for the entire duration of the study, covering its own civil liability as well as that of all the doctors involved in the realization of the study. It will also insure the full compensation for harmful consequences of the research for the participating persons and their beneficiaries, except with evidence, at their responsibility, that the damage is not attributable to their mistake or to that of all consultants, without the possibility of being opposed to an act by a third party or the voluntary withdrawal of the person who had initially consented to participate in the research.

The insurance contract was signed before the start of the study with the *Société Hospitalière d’Assurance Mutuelle*, 18 rue Edouard Rochet, 69008 Lyon, under the number 159077.

During the research involving human individuals or at its end, the data collected on the persons participating and sent to the sponsor by the investigators (or any other specialists) will be made anonymous. The persons having direct access to the data will take all necessary precautions to ensure the confidentiality of the information related to the trials, to the persons participating and, in particular, with regard to their identity as well as the results obtained.

In the event that a substantial modification is made to the protocol by the investigator, it will be approved by the sponsor. Before its implementation, the latter must obtain a favorable opinion from the EC and an authorization from the ANSM within the scope of their respective competencies. The coordinating investigator and coordinating center will then notify the other centers and the sponsor will update the protocol in the clinical trial registry.

### Dissemination of the results

The protocol of the study will be published during the trial. The results will be processed within 1-year of the last visit of last patient. The results will be published in a peer-reviewed journal, recorded in accordance with the Consolidated Standards of Reporting Trials (CONSORT) Statement. Patients will be informed of global and individual (treatment arm, biological data) results of the study.

## Discussion

Urinary calcium is an important intermediate criterion in the daily management of patients with nephrolithiasis associated with hypercalciuria and increased 1,25(OH)_2_D levels: normalization and decrease of urinary calcium can prevent/slow down the progression of nephrocalcinosis/ nephrolithiasis, and therefore CKD and bone complications occurring during adulthood.

Measures focused on modifying risk factors (sodium intake, hyperhydration, potassium citrate, and hydrochlorothiazide) have low impact on hypercalciuria. Thus, the current challenge is to propose an efficient treatment in order to prevent later complications and notably the development of CKD that can ultimately lead to end-stage renal disease, dialysis, and renal transplantation. Based on improvement of physiopathology and genetic knowledge in phosphate/calcium metabolism, the “off-label” use of fluconazole was recently reported to be useful in hypercalciuric patients with increased 1,25(OH)_2_D levels. The pharmacokinetic and tolerability are well known from anti-infectious indications.

Thus, the FLUCOLITH trial is a unique opportunity to develop a new indication of a well-known and not expensive drug (e.g., fluconazole) in rare renal diseases, the ultimate objective being the secondary prevention of CKD worsening in these patients.

If the results of this proof-of-concept randomized controlled trial are positive, we will propose an extension phase to evaluate the long-term efficacy and safety of fluconazole on renal and bone parameters.

## Trial status

The FLUCOLITH study is currently recruiting patients. Inclusions began on 13 January 2021. The estimated inclusion period is 36 months.

## Supplementary Information

**Additional file 1.** Spirit figure: schedule of enrolment, interventions, and assessments of the FLUCOLITH trial

**Additional file 2.** List of study sites.

**Additional file 3.** DSMB Charter.

**Additional file 4.** FLUCOLITH_SPIRIT-Checklist.

**Additional file 5.** Flucolith_Manuel laboratoire_V5R1.

**Additional file 6.** NIFC_Adultes_V4_20210322_FLUCOLITH smaR1.

## Data Availability

At the end of the study, data will be available in the study database which is not publicly available. They cannot be provided until the end of the study but will be available at the end, from the corresponding author, after approval of the sponsor, and after thorough review of the scientific interest of the request by the study team. All study data of the study is the property of the HCL Hospital.
