# Supplementary material for: Fluconazole in hypercalciuric patients with increased 1,25(OH)2D levels: the prospective, randomized, placebo-controlled, double-blind FLUCOLITH trial
Source: Trials. 2022 Jun 16;23:499. doi: 10.1186/s13063-022-06302-z (PMC9204961; doi:10.1186/s13063-022-06302-z)
Supplement: Supplementary file 1 — Additional file 1. Spirit figure: schedule of enrolment, interventions, and assessments of the FLUCOLITH trial [file 13063_2022_6302_MOESM1_ESM.docx]

|  | **STUDY PERIOD** | | | | | | | | | |  |
| --- | --- | --- | --- | --- | --- | --- | --- | --- | --- | --- | --- |
|  | **Enrolment** | **randomization** | **Titration period** | | | **Stable period** | | | | **safety** | |
| **TIMEPOINT**** | ***V1*** | **V2** | ***V3*** | ***V4*** | ***V5*** | ***V6*** | ***V7*** | ***V8*** | ***V9*** | ***V10*** | |
| **ENROLMENT:** |  |  |  |  |  |  |  |  |  |  | |
| Eligibility screen | X |  |  |  |  |  |  |  |  |  | |
| Informed consent | X |  |  |  |  |  |  |  |  |  | |
| Allocation |  | X |  |  |  |  |  |  |  |  | |
| **INTERVENTIONS:** |  |  |  |  |  |  |  |  |  |  | |
| *[Placebo]* |  |  |  |  |  |  |  |  |  |  | |
| *[Fluconazole]* |  |  |  |  |  |  |  |  |  |  | |
| **ASSESSMENTS:** |  |  |  |  |  |  |  |  |  |  | |
| *[Clinical examination]* | x | x |  | x |  | x |  | x | x |  | |
| *[Treatment dispensation]* |  | x |  | x |  | x |  | x |  |  | |
| *[Compliance]* |  |  |  | x |  | x | x | x | x |  | |
| *[24-hour urinary analysis]* | x |  | x | x | x | x |  | x | x |  | |
| *[Urinary pregnancy test]* | x | x |  | x |  | x | x | x | x |  | |
| *[blood analyses]* | x | x | x | x | x | x | x | x | x | x | |
| *[biocollection : serum, plasma, urines]* |  | x |  |  |  |  |  |  | x |  | |
| *[Oral calcium load test]* |  | x |  |  |  |  |  |  | x |  | |
| *[Quality of life questionnaire]* |  | x |  |  |  |  |  |  | x |  | |
| *[Treatment satisfactory questionnaire]* |  |  |  |  |  |  |  |  | x |  | |
| *[standardized dietetic consultation]* |  | x |  |  |  |  |  |  | x |  | |
| *[electrocardiogram]* | x |  |  | x |  | x |  | x |  |  | |
| *[Renal ultrasound]* |  | x |  |  |  |  |  | x |  |  | |
| *[* *Dual X-ray absorptiometry]* |  | x |  |  |  |  |  |  |  |  | |
| *[Urine and buccal microbiological samples]* |  | x |  |  |  | x |  | x |  |  | |
| *[Genetic analysis]* |  | x |  |  |  |  |  |  |  |  | |
| *[Adverse events]* | x | x | x | x | x | x | x | x | x | x | |
| *[Concomitant medications]* | x | x | x | x | x | x | x | x | x |  | |
