# Supplementary material for: Fluconazole in hypercalciuric patients with increased 1,25(OH)2D levels: the prospective, randomized, placebo-controlled, double-blind FLUCOLITH trial
Source: Trials. 2022 Jun 16;23:499. doi: 10.1186/s13063-022-06302-z (PMC9204961; doi:10.1186/s13063-022-06302-z)
Supplement: Supplementary file 2 — Additional file 2. List of study sites. [file 13063_2022_6302_MOESM2_ESM.docx]

**01 - Dr Aurélia BERTHOLET-THOMAS**

Service de Néphrologie Rhumatologie Dermatologie Pédiatrique

Hospices Civils de Lyon – Groupement Hospitalier Est

Hôpital Femme Mère Enfant

59 Bb Bd Pinel – 69677 Bron

Tél : 04 27 85 61 28 - Email : [aurelia.bertholet-thomas@chu-lyon.fr](mailto:aurelia.bertholet-thomas@chu-lyon.fr)

**02 - Dr Sandrine LEMOINE**

Service d’Exploration fonctionnelle rénale

Hôpital Edouard Herriot

5 place d’Arsonval - 69003 Lyon

Tél : 04 72 11 02 52 - Email: [sandrine.lemoine@chu-lyon.fr](mailto:sandrine.lemoine@chu-lyon.fr)

**03 - Pr Jérôme HARAMBAT**

Service de Néphropédiatrie

CHU Pellegrin Hôpital des Enfants

Place Amélie Raba-Léon

330768 Bordeaux

Tél : 05 57 82 28 2805 56 79 87 25 - Email : [jerome.harambat@chu-bordeaux.fr](mailto:jerome.harambat@chu-bordeaux.fr)

**04 - Dr Philippe ECKART**

Service de Pédiatrie médicale - Néphrologie

CHU de Caen - Hôpital de la Côte de Nacre

Avenue de la Côte de Nacre - 14033 Caen Cedex 9

Tél: 02 31 27 25 87 - Email : [eckart-p@chu-caen.fr](mailto:eckart-p@chu-caen.fr)

**05 – Dr Gilbert ZANETTA**

CHU de Dijon

Service de Soins intensifs de Néphrologie

14 rue Paul Gaffarel – 21079 Dijon Cedex

Tél. : 03.80.29.34.34 – Email : [gilbert.zanetta@chu-dijon.fr](mailto:gilbert.zanetta@chu-dijon.fr)

**06 – Dr Guylhène BOURDAT-MICHEL**

Service de Pédiatrie - Néphrologie pédiatrique

Pôle Couple Enfant

CHU Grenoble Alpes - Site Nord - Hôpital Couple Enfant

Boulevard de la Chantourne 38043 Grenoble Cedex 9

Tél : 04 76 76 58 94 - Email : [Gbourdatmichel@chu-grenoble.fr](mailto:Gbourdatmichel@chu-grenoble.fr)

**07 – Dr Marc FILA**

Service de néphrologie pédiatrique

CHU Arnaud de Villeneuve

371 avenue du Doyen Gaston Giraud - 34295 Montpellier Cedex 5

Tel: 04 67 33 91 70 - Email : [m-fila@chu-montpellier.fr](mailto:m-fila@chu-montpellier.fr)

**08 – Dr Guillaume FAVRE**

Service de Néphrologie-Dialyses-Transplantation

Hôpital Pasteur – CHU de Nice

30 Voie Romaine, BP 1069 - 06002 Nice

Email : [favre.g@chu-nice.fr](mailto:favre.g@chu-nice.fr)

**09 - Dr Lucile FIGUERES**

Service de Néphrologie et Immunologie Clinique

CHU de Nantes

1 Place Alexis Ricordeau - 44000 Nantes

Tél : 02 40 08 73 2341 97 - Email : [lucile.figueres@chu-nantes.fr](mailto:lucile.figueres@chu-nantes.fr)

**10 - Dr Véronique BAUDOUIN**

Service de Néphrologie Pédiatrique

Hôpital Robert Debré

48 boulevard Sérurier - 75019 Paris

Tél : 01 40 03 47 82 - Email : [veronique.baudouin@aphp.fr](mailto:veronique.baudouin@aphp.fr)

**11 - Pr. Olivia GILLION BOYER**

Service de Néphrologie Pédiatrique

Hôpital Universitaire Necker-Enfants Malades

149 rue de Sèvres - 75743 Paris Cedex 15

Tél : 01 44 49 44 63 - Email : [olivia.boyer@aphp.fr](mailto:olivia.boyer@aphp.fr)

**12 - Pr. Bertrand KNEBELMANN**

Service de Néphrologie-Dialyse Adultes

Hôpital Universitaire Necker

149 rue de Sèvres

75743 Paris Cedex 15

Tél. : 01 44 49 54 58 - Email : [bertrand.knebelmann@aphp.fr](mailto:bertrand.knebelmann@aphp.fr)

**13 - Pr. Emmanuel LETAVERNIER**

Service d’Explorations fonctionnelles multidisciplinaires

Hôpital Tenon

4 rue de la Chine - 75020 Paris

Tél. : 01 56 01 67 73 - Email : [emmanuel.letavernier@aphp.fr](mailto:emmanuel.letavernier@aphp.fr)

**14 – Dr Anne BLANCHARD**

Service de Néphrologie et d’Hémodyalise

APHP - Hôpital Européen Georges Pompidou HEGP

20 rue Leblanc – 75015 Paris

Tél.: 01 56 09 20 72 - Email : [anne.blanchard@aphp.fr](mailto:anne.blanchard@aphp.fr)

**15 – Pr. Agnès LINGLART**

Service d'Endocrinologie et Diabète de l'Enfant

CHU Paris-Sud - Hôpital de Bicêtre

78 rue du Général Leclerc - 94270 Le Kremlin-Bicêtre

Tél : 01 45 21 78 31 - Email : [agnes.linglart@aphp.fr](mailto:agnes.linglart@aphp.fr)

**16 - Dr Thomas STEHLE**

Service de Néphrologie et transplantation

Hôpital Henri Mondor

51, avenue du Mal de Lattre de Tassigny - 94010 Créteil cedex

Tél : 01 49 81 24 57 - Email : [thomas.stehle@aphp.fr](mailto:thomas.stehle@aphp.fr)

**17 – Pr. Christine PIETREMENT**

Service de Pédiatrie générale et spécialisée

Pôle Femme-Parents-Enfant

Centre hospitalier universitaire de Reims

49 rue Cognacq Jay - 51100 Reims

Tél: 03 26 78 7007 - Email: [cpietrement@chu-reims.fr](mailto:cpietrement@chu-reims.fr)

**18 - Dr Amélie RICKEWAERT**

Service de Néphrologie pédiatrique

CHU Hôpital Sud

16 bd Bulgarie - 35203 Rennes Cedex 2

Tél : 02 99 26 71 14 - Email : [amelie.ryckewaert@chu-rennes.fr](mailto:amelie.ryckewaert@chu-rennes.fr)

**19 – Dr Henri BRENIER**

Service de Néphrologie

CHU Rennes Pontchaillou

2 rue Henri le Guilloux – 35000 Rennes

Tél. : 02 98 28 43 21 – Email : [henri.brenier@chu-rennes.fr](mailto:henri.brenier@chu-rennes.fr)

**20 - Dr Françoise BROUX**

Unité Néphro-Hémodialyse Pédiatrique/Hôpital de Jour

Hôpital Charles Nicolle - CHU de Rouen

1, rue de Germont - 76031 Rouen cedex

Tél : 02 32 88 85 43 - Email : [francoise.broux@chu-rouen.fr](mailto:francoise.broux@chu-rouen.fr)

**21 - Dr Dominique GUERROT**

Service de Néphrologie

CHU Hôpitaux de Rouen

1 rue de Germont - 76031 Rouen

Tél : 02 32 88 90 15 - Email : [dominique.guerrot@chu-rouen.fr](mailto:dominique.guerrot@chu-rouen.fr)

**22 –Dr Krummel Thierry**

Service d’endocrinologie, diabète et nutrition

Hôpital Civil

Clinique Médicale B

1 place de l’Hôpital - 67091 STRASBOURG cedex

Tél.: 03 88 11 66 03 - Email : [thierry.krummel@chru-strasbourg.fr](mailto:thierry.krummel@chru-strasbourg.fr)

**23 - Dr Ariane ZALOSZYC**

Service de Néphrologie Pédiatrique

CHU de Strasbourg, hôpital de Hautepierre

1 Avenue Moliere - 67000 Strasbourg

Tél : 03 88 12 80 0077 42 - Email : [ariane.zaloszyc@chru-strasbourg.fr](mailto:ariane.zaloszyc@chru-strasbourg.fr)

**24 - Dr Marc PADILLA**

Service de Néphrologie

CHU Grenoble Alpes - Site Nord

Boulevard de la Chantourne 38043 Grenoble Cedex 9

Tél : 04 76 76 54 60 - Email : [mpadilla@chu-grenoble.fr](mailto:mpadilla@chu-grenoble.fr)

**25 - Dr François GLOWACKI**

Service de Néphrologie

CHU de Lille – Hôpital Huriez

Rue Michel Polonowski - 59037 Lille

Tél : 03 20 44 40 75 - Email : [francois.glowacki@chru-lille.fr](mailto:francois.glowacki@chru-lille.fr)

**26 - Pr Jean-Pierre SALLES**

Service de Pédiatrie Endocrinologie

CHU de Toulouse – Hôpital des Enfants

330, avenue de Grande Bretagne - TSA 70034 - 31059 Toulouse cedex 9

Tél : 05 67 77 13 05 - Email : [salles.jp@chu-toulouse.fr](mailto:salles.jp@chu-toulouse.fr)
