# Supplementary material for: Fluconazole in hypercalciuric patients with increased 1,25(OH)2D levels: the prospective, randomized, placebo-controlled, double-blind FLUCOLITH trial
Source: Trials. 2022 Jun 16;23:499. doi: 10.1186/s13063-022-06302-z (PMC9204961; doi:10.1186/s13063-022-06302-z)
Supplement: Supplementary file 3 — Additional file 3. DSMB Charter. [file 13063_2022_6302_MOESM3_ESM.pdf]

HCL<br>Direction de la Recherche Clinique et de l'Innovation	**Independent Data Safety Monitoring Board Charter**

**Protocol Title :**	Fluconazole as a new therapeutic tool in hypercalciuric patients with increased 1,25(OH)2D levels - The FLUCOLITH study
**Type of study:**	Phase II, research involving the human person (RIPH) category 1 (interventional study)
**Protocol references :**	Sponsor Reference: 69HCL20_0071<br>**N°EudraCT:** 2020-003011-97
**Coordinating investigator:**	Dr Aurélia BERTHOLET-THOMAS
**Sponsor :**	**Hospices Civils de Lyon**<br>BP 2251<br>3 Quai des Célestins<br>69229 LYON Cedex 02<br>FRANCE
**Protocol Version :**	V3.0 of 23/11/2020

1 Introduction

The charter defines the Data Safety Monitoring Board (DSMB), its role and its responsibilities, its goals, the relations with the other clinical research participants and the meeting timings. It defines as well, the procedures which insure the confidentiality, the communication modalities and the respect of methodology and statistical guidelines.

Responsibilities of DSMB

- Protection of the rights, safety and well-being of human subjects involved in the trial
- Safety and efficacy evaluation of study procedures
- Ensure that study is properly conducted and that study procedures are followed

The DSMB must with the transmitted data:

- Review the individual participant data or the analysis results from a safety point of view in order to evaluate continuously the balance benefit/risk of the study.

- Monitor the study design, in particular: the implemented procedures for the adverse events in order to maximize the participant's safety in the study and minimize the risks; and the data which allow the proper functioning of the DSMB.

- Analyze the results of at least 5 of the first adults exposed to the treatment after the V_8 visit (10 weeks of stable treatment after the titration period). .Its analysis will be transmit to the ANSM in the form of Substantial Modification for Authorization (SMA)before allowing the inclusion of minor patients (less than 18 years old).

- Evaluate the study progress by assessing the balance benefit/risk of patients periodically and other factors which can influence the study results.

- Take into account study external factors, such as scientific or therapeutic developments which can have an impact on the participant's safety or the study's ethic.

- Examine the documentation about the serious adverse events and the safety reports.

- Evaluate and report to the coordinating investigator and the sponsor any problem which can impact the proper conduct of the study, the participant's recruitment, the sample size and/or the data collection.

- Establish recommendations to the study sponsor for the continuation, the interruption or other modifications about the study, based on the accumulated experience, including the observed, favorable or adverse effects of the study treatment.

The committee must identify any elements which could influence on the protection of the rights, safety and well-being of the participants of the study and the good overall conduct of the study.

2 Organisation

2.1 Composition

The DSMB is an independent (which do not participate to the study and work at HCL), advisory and multidisciplinary group, composed of:

- A clinical medical specialist in nephrolithiasis: Fernando SANTOS, nephro-pediatrician, Oviedo, Asturia, Espagne.
- A second specialist in nephrolithiasis: Yvan TACK, adult nephrologist, Toulouse, France ;
- A methodologist: Ricardo FERNANDEZ, pediatrician and pharmacologist, Lisbon, Portugal ;
- And an infectologist : Julien SAISON, infectiologist, Valence, France.

who have collectively an experience in the management and the conduct of clinical studies.
The DSMB members are approved by the sponsor and the coordinating investigator. The DSMB members are appointed/nominated for the study duration. In case a member cannot continue to participate in the DSMB, the sponsor can substitute the member according to the study progress and the relevance.
NB: The DSMB can be a source of proposal for this substitution.

2.2 Biostatistician participation

Ideally, the biostatistician who presents the report to the DSMB members is a biostatistician independent from the study. In case of impossibility, the study's biostatistician will give the needed information and the closed session report to the DSMB members. The biostatistician can participate to the closed session of the DSMB in order to facilitate the results presentation to the DSMB members, but by no means participate in the vote since he is not a DSMB member.

2.3 Conflicts of interest

The DSMB members must sign a declaration of conflicts of interest (last page on the charter):
They must not have significant conflicts of interest (on financial or scientific issues) with the study. In that respect, neither the study investigators, the sponsor employed persons nor the persons which may have relate to the study treatment cannot be a DSMB member.
The DSMB members must not have shares from the company whose treatment is being assessed in the study. Any member of the DSMB who acquires important conflicts of interest during the study must resign from the DSMB.

3 DSMB meetings

3.1 Meetings conditions

The sponsor is responsible for the organization and the DSMB management. It can delegate the management to the study coordination center which will be then the principal interlocutor for the DSMB.
Any other person (except the sponsor or its representative) involved in the study must not communicate directly with the DSMB members about the study, apart from the opened session.

The person / the biostatistician performing the analyses and presenting the report to the DSMB members will be responsible for the information exchanges with the DSMB. This person will centralize the closed session report (statistical and safety report) and will be the only one to transmit this information (aggregated or per arm, under a semi-unblinding or opened) to the DSMB members. The study integrity will thus be respected.

The DSMB meetings may be held in person or by teleconference and actions will be taken to assure that only the appropriate persons participate in the opened or closed sessions. If a member of the DSMB cannot be present at one of the meeting like initially scheduled, this member should immediately inform the sponsor. In the absence of one of the members during the meeting 0, the chairman will determine the notice's validity. Without a named chairman, the experts will communicate with each other (mail, teleconference or other) to transmit the final notice.

The meetings frequency will be defined during the first meeting. The date, the hour and the DSMB meeting calendar are determined by mutual agreement between the DSMB members and the sponsor (or the coordination center). Additional meetings can be requested by the DSMB according to the information received during the study by the sponsor, in particular in case of:

- Increased of expected adverse reactions or serious adverse reactions frequency;
- Increased of unexpected serious adverse reactions.

If the coordinating investigator wants to request the DSMB for an exceptional meeting, the investigator should ask the sponsor or the structure responsible for the DSMB management.

3.2 First meeting or meeting 0 :

The aim of the first DSMB meeting is to:

- ✓ Remind the DSMB role and the members role, underline the importance of respecting the data integrity
- ✓ Be aware of the study and possibly give recommendations about the protocol (design, objectives, collected data ...) including the study and treatment interruption criteria.
- ✓ Provide recommendations about safety data which should be particularly monitored by the sponsor.
- ✓ Validate the standard report format which should be supplied by the sponsor to the DSMB: particularly define the data that the DSMB wishes to receive and the need for introducing the data under a semi-unblinding, etc ...
- ✓ Elect a chairman (signatory of the DSMB notice)
- ✓ Define and validate the meeting frequency and the communication modalities

3.3 Data review meetings

These meetings include 2 sessions :

- An opened session with DSMB members, the sponsor, the coordinating investigator (or the representative), the coordination, the study methodologist, the biostatistician who performs the analyses for the DSMB, the safety unit and if necessary any other study participant (datamanager, biologist, ...). The aim of this session is to facilitate the interactions, the discussions between the DSMB members and the study staff and to provide the information which can impact their decision. No comparative data (blinding or unblinding) must be presented or discussed during this session.

- A closed session restricted to the DSMB members and the biostatistician/person who performed the analyses for the DSMB (non-voting) only. The aim of this session is to determine a consensus and the DSMB member's recommendations to the study sponsor. The DSMB members must not disclose any comparative information about the study patients to the study staff during their recommendations.

4 The reports

The reports must be forwarded to the DSMB members at least **a week** before the date of the meeting.

4.1 DSMB reports content

Two different reports can be distinguished:

- **An opened session report** including data about the study progress (inclusions ...), study quality data (deviation, experienced problems, missing data about the evaluation criteria, etc ...), administrative and regulatory data (amendments, monitoring summary ...).
  All these information will be transmitted to the opened session participants before the DSMB meeting by the sponsor or the coordination center if applicable.
- **A closed session report** including a detailed section about the safety data (tolerance data, SAE, AE if applicable ...), statistical report (demographic data, security and efficiency data if applicable ...) in the format defined at the meeting 0.
  All these information (which can be aggregated or per arm, under a semi-unblinding or opened etc...) will be transmitted to the members only, before the DSMB closed session by the biostatistician/person who performs the DSMB analysis. No other person involved in the study must have an access to the data in order to preserve the study integrity and the results.

If necessary, the DSMB can ask additional information to the sponsor.
For this controlled double-blind trial, the safety data will be presented in a semi-unblinding (A arm/ B arm), in accordance with the statistician, excepting a specific DSMB request.

4.2 Recommendations made by the DSMB

After each meeting, the DSMB will give recommendations to the sponsor about the study pursuit with possible protocol amendments and about the management of the study participants.

The recommendations will be sent to the sponsor **the week following the meeting** by the DSMB chairman chosen during the meeting 0.
The DSMB notice is consultative. Upon receiving the DSMB recommendations, the sponsor will make its decision and will inform the DSMB if its opinion differs.

The sponsor or the coordination center will transmit to the coordinating investigator the DSMB recommendations and the sponsor decisions if different.

4.3 Closed session meeting report

The closed session meeting report: it complements the recommendations but may include discussion elements between the members, which could compromise the study integrity. Therefore, this report must remain confidential.
If necessary, the DSMB chairman should write a confidential report describing the exchanges of the closed session. This confidential report should be retained by the DSMB chairman during the time of the study and addressed to the sponsor at the end of the study for archiving, after the final analysis. It should be available during the time of the study if requested by the authorities or the sponsor.

Secretariat telephone:
....
Email:
....
Mobile phone :
....

Acceptance of the DSMB charter

Please send by mail at sa.drci.vigilance@chu-lyon.fr this form to the sponsor AND send the original documents by letter to the administrative offices of the HCL at: Siège administratif des HCL, DRCI, Vigilance des essais, 3 quai des célestins, 69002 LYON – FRANCE.

I, [illegible] (*specify your first name and last name*), Data Safety Monitoring Board (DSMB) member, for the FLUCOLITH study, agree with the terms of this charter V3.0 of 23/11/2020. If the charter is significantly changed, all the DSMB members will review the modifications and will give approval of the new charter.

Declaration of conflict of interest

I, [illegible] (*specify your first name and last name*), DSMB member as a [illegible] (specify your specialty) expert for the FLUCOLITH study, confirm agreement with the following points:

I will undertake to :

- Protect the rights, safety and well-being of human subjects involved in the trial;
- Preserve the study integrity ;
- Be free from preconceived ideas and prejudices;
- Respect scientific and ethical standards, and eliminate or divulge real or apparent conflicts of interest during my participation to the clinical study.

Furthermore:

I declare that my spouse or my children, my employer or I have no financial interest in the study.

I undertake not to interfere with the analysis or the publication of the study results.

I undertake not to have a part-time work or a full time work, paid or unpaid in all the organizations which are : (a) involved in the study; (b) whose the products will be used or tested in the study, or whose the products or services will be directly and predictably importantly affected by the study results;

I undertake not being a leader, member, owner, fiduciary, administrator, expert or consultant of these organizations.

Confidentiality

I understand that pursuant to the Article R. 5121-13 of the French Public Health Code, I undertake not to disclose the scientific or technical confidential information which belong to the sponsor (mentioned below as "confidential information") coming to my knowledge during my DSMB participation.

I undertake, during the study and for five (5) years after the end of the study for any reason whatsoever, to:

- Protect, keep strictly confidential and process with degree of caution and protection, at least equal to the degree that I allow to my own confidential information, all the confidential information which I could know ;
- Not divulge the confidential information, directly or indirectly, to any third party ;
- Not use the confidential information, fully or partially, for a purpose other than the purpose defined in this charter without the prior and written consent of the sponsor ;
- Not request an industrial property title or exercise intellectual property rights or any other rights about confidential information communicated under this current DSMB;
- Not copy, reproduce or duplicate the confidential information, partially or completely, when such copies, reproductions or duplications were not approved by the sponsor.

Confidentiality obligations will apply only to the confidential data which will be expressly granted as confidential by the sponsor during their release in whatever form.
For this purpose, the sponsor will transmit confidential data with the mention CONFIDENTIAL on the hardware support of the divulged information.
Without hardware support, the sponsor undertakes to expressly inform you about the confidentiality nature of the information and will confirm you the confidential nature in writing as soon as possible and not later than 30 days after their release.

Any confidential data and their reproductions transmitted by the sponsor to your attention will stay the sponsor property and should be restitute immediately to the sponsor upon request.

Except as provided above, you will have no obligation and will not be subject to any restriction with regard to all confidential data which you will be able to demonstrate:

- That they have entered the public domain prior to their disclosure or after it, but in this case without any fault attributable to you; or
- That they are already known; this previous knowledge being able to be demonstrated by the existence of appropriated documents in your records; or
- That they have been sent to you by third party in a lawful manner, without restriction or violation of this charter; or
- That they have been published without contravening the terms of this charter; or
- That they have not been designated or confirmed as confidential data in the present charter.

It is expressly agreed that the release and the provision of confidential data by the sponsor under this Charter cannot be under any circumstances interpreted as conferring to you any right (under a license or by some other means) expressly or implied about topics, inventions or discoveries to which these confidential data relate.

The same is true for the authors 'rights or other rights related to the literary and artistic property, trademarks or trade secrets.

Furthermore, it is agreed that the property right on all confidential data that sponsor will disclose to you under this Charter belongs in any case to the sponsor, respecting the rights of third parties.

I will receive information from the Hospices Civils de Lyon, the study sponsor, including exclusive and confidential information.

I understand that I will have an access to records in order to participate to the study DSMB.
In my current role of DSMB member as expert, I will not publish or reproduce these documents under any circumstances.
I recognize that I do not use these documents except for my missions as a study member.

I shall take all necessary precautions to prevent any unauthorized person from having access to these records.
I shall keep all documentation confidential up to the end of the study.
I have read all the terms and conditions of this charter and I am committing to these terms and conditions.

[illegible] [illegible] 1|3|-|1|0|-|2|0|2|1|

Signature First name and last name Date

Secretariat telephone:
05 61 32 26 83
Email:
ivan.tack@univ-tlse3.fr
Mobile phone :
06-18-64-23-31

Acceptance of the DSMB charter

Please send by mail at sa.drci.vigilance@chu-lyon.fr this form to the sponsor AND send the original documents by letter to the administrative offices of the HCL at: Siège administratif des HCL, DRCI, Vigilance des essais, 3 quai des célestins, 69002 LYON – FRANCE.

I, IVAN TACK (specify your first name and last name), Data Safety Monitoring Board (DSMB) member, for the FLUCOLITH study, agree with the terms of this charter V3.0 of 23/11/2020. If the charter is significantly changed, all the DSMB members will review the modifications and will give approval of the new charter.

Declaration of conflict of interest

I, IVAN TACK (specify your first name and last name), DSMB member as a Adult Nephrologist (specify your specialty) expert for the FLUCOLITH study, confirm agreement with the following points:

I will undertake to :

- Protect the rights, safety and well-being of human subjects involved in the trial;
- Preserve the study integrity ;
- Be free from preconceived ideas and prejudices;
- Respect scientific and ethical standards, and eliminate or divulge real or apparent conflicts of interest during my participation to the clinical study.

Furthermore:

I declare that my spouse or my children, my employer or I have no financial interest in the study.

I undertake not to interfere with the analysis or the publication of the study results.

I undertake not to have a part-time work or a full time work, paid or unpaid in all the organizations which are : (a) involved in the study; (b) whose the products will be used or tested in the study, or whose the products or services will be directly and predictably importantly affected by the study results;

I undertake not being a leader, member, owner, fiduciary, administrator, expert or consultant of these organizations.

Confidentiality

I understand that pursuant to the Article R. 5121-13 of the French Public Health Code, I undertake not to disclose the scientific or technical confidential information which belong to the sponsor (mentioned below as "confidential information") coming to my knowledge during my DSMB participation.

I undertake, during the study and for five (5) years after the end of the study for any reason whatsoever, to:

- Protect, keep strictly confidential and process with degree of caution and protection, at least equal to the degree that I allow to my own confidential information, all the confidential information which I could know ;
- Not divulge the confidential information, directly or indirectly, to any third party ;
- Not use the confidential information, fully or partially, for a purpose other than the purpose defined in this charter without the prior and written consent of the sponsor ;
- Not request an industrial property title or exercise intellectual property rights or any other rights about confidential information communicated under this current DSMB;
- Not copy, reproduce or duplicate the confidential information, partially or completely, when such copies, reproductions or duplications were not approved by the sponsor.

Confidentiality obligations will apply only to the confidential data which will be expressly granted as confidential by the sponsor during their release in whatever form.

For this purpose, the sponsor will transmit confidential data with the mention CONFIDENTIAL on the hardware support of the divulged information.

Without hardware support, the sponsor undertakes to expressly inform you about the confidentiality nature of the information and will confirm you the confidential nature in writing as soon as possible and not later than 30 days after their release.

Any confidential data and their reproductions transmitted by the sponsor to your attention will stay the sponsor property and should be restitute immediately to the sponsor upon request.

Except as provided above, you will have no obligation and will not be subject to any restriction with regard to all confidential data which you will be able to demonstrate:

- That they have entered the public domain prior to their disclosure or after it, but in this case without any fault attributable to you; or
- That they are already known; this previous knowledge being able to be demonstrated by the existence of appropriated documents in your records; or
- That they have been sent to you by third party in a lawful manner, without restriction or violation of this charter; or
- That they have been published without contravening the terms of this charter; or
- That they have not been designated or confirmed as confidential data in the present charter.

It is expressly agreed that the release and the provision of confidential data by the sponsor under this Charter cannot be under any circumstances interpreted as conferring to you any right (under a license or by some other means) expressly or implied about topics, inventions or discoveries to which these confidential data relate.

The same is true for the authors 'rights or other rights related to the literary and artistic property, trademarks or trade secrets.

Furthermore, it is agreed that the property right on all confidential data that sponsor will disclose to you under this Charter belongs in any case to the sponsor, respecting the rights of third parties.

I will receive information from the Hospices Civils de Lyon, the study sponsor, including exclusive and confidential information.

I understand that I will have an access to records in order to participate to the study DSMB.
In my current role of DSMB member as expert, I will not publish or reproduce these documents under any circumstances.
I recognize that I do not use these documents except for my missions as a study member.

I shall take all necessary precautions to prevent any unauthorized person from having access to these records.
I shall keep all documentation confidential up to the end of the study.
I have read all the terms and conditions of this charter and I am committing to these terms and conditions.

Signature	Ivan TACK	30-12-1962
Signature	First name and last name	Date

CENTRE HOSPITALIER DE VALENCE
Maladies Infectieuses
Dr Julien SAISON
N° RPPS : 10100[illegible]

Secretariat telephone:

……………………………

Email: jsaison@ch-valence.fr

Mobile phone : 06 76 10 67 [illegible]

Acceptance of the DSMB charter

Please send by mail at sa.drci.vigilance@chu-lyon.fr this form to the sponsor AND send the original documents by letter to the administrative offices of the HCL at: Siège administratif des HCL, DRCI, Vigilance des essais, 3 quai des célestins, 69002 LYON – FRANCE.

I, Julien SAISON *(specify your first name and last name)*, Data Safety Monitoring Board (DSMB) member, for the FLUCOLITH study, agree with the terms of this charter V3.0 of 23/11/2020. If the charter is significantly changed, all the DSMB members will review the modifications and will give approval of the new charter.

Declaration of conflict of interest

I, Julien SAISON *(specify your first name and last name)*, DSMB member as a ______________________ (specify your specialty) expert for the FLUCOLITH study, confirm agreement with the following points:

I will undertake to :

- Protect the rights, safety and well-being of human subjects involved in the trial;
- Preserve the study integrity ;
- Be free from preconceived ideas and prejudices;
- Respect scientific and ethical standards, and eliminate or divulge real or apparent conflicts of interest during my participation to the clinical study.

Furthermore:

I declare that my spouse or my children, my employer or I have no financial interest in the study.

I undertake not to interfere with the analysis or the publication of the study results.

I undertake not to have a part-time work or a full time work, paid or unpaid in all the organizations which are : (a) involved in the study; (b) whose the products will be used or tested in the study, or whose the products or services will be directly and predictably importantly affected by the study results;

I undertake not being a leader, member, owner, fiduciary, administrator, expert or consultant of these organizations.

Confidentiality

I understand that pursuant to the Article R. 5121-13 of the French Public Health Code, I undertake not to disclose the scientific or technical confidential information which belong to the sponsor (mentioned below as "confidential information") coming to my knowledge during my DSMB participation.

I undertake, during the study and for five (5) years after the end of the study for any reason whatsoever, to:

- Protect, keep strictly confidential and process with degree of caution and protection, at least equal to the degree that I allow to my own confidential information, all the confidential information which I could know ;
- Not divulge the confidential information, directly or indirectly, to any third party ;
- Not use the confidential information, fully or partially, for a purpose other than the purpose defined in this charter without the prior and written consent of the sponsor ;
- Not request an industrial property title or exercise intellectual property rights or any other rights about confidential information communicated under this current DSMB;
- Not copy, reproduce or duplicate the confidential information, partially or completely, when such copies, reproductions or duplications were not approved by the sponsor.

Confidentiality obligations will apply only to the confidential data which will be expressly granted as confidential by the sponsor during their release in whatever form.

For this purpose, the sponsor will transmit confidential data with the mention CONFIDENTIAL on the hardware support of the divulged information.

Without hardware support, the sponsor undertakes to expressly inform you about the confidentiality nature of the information and will confirm you the confidential nature in writing as soon as possible and not later than 30 days after their release.

JS

Any confidential data and their reproductions transmitted by the sponsor to your attention will stay the sponsor property and should be restitute immediately to the sponsor upon request.

Except as provided above, you will have no obligation and will not be subject to any restriction with regard to all confidential data which you will be able to demonstrate:

- That they have entered the public domain prior to their disclosure or after it, but in this case without any fault attributable to you; or
- That they are already known; this previous knowledge being able to be demonstrated by the existence of appropriated documents in your records; or
- That they have been sent to you by third party in a lawful manner, without restriction or violation of this charter; or
- That they have been published without contravening the terms of this charter; or
- That they have not been designated or confirmed as confidential data in the present charter.

It is expressly agreed that the release and the provision of confidential data by the sponsor under this Charter cannot be under any circumstances interpreted as conferring to you any right (under a license or by some other means) expressly or implied about topics, inventions or discoveries to which these confidential data relate.

The same is true for the authors 'rights or other rights related to the literary and artistic property, trademarks or trade secrets.

Furthermore, it is agreed that the property right on all confidential data that sponsor will disclose to you under this Charter belongs in any case to the sponsor, respecting the rights of third parties.

I will receive information from the Hospices Civils de Lyon, the study sponsor, including exclusive and confidential information.

I understand that I will have an access to records in order to participate to the study DSMB.
In my current role of DSMB member as expert, I will not publish or reproduce these documents under any circumstances.
I recognize that I do not use these documents except for my missions as a study member.

I shall take all necessary precautions to prevent any unauthorized person from having access to these records.
I shall keep all documentation confidential up to the end of the study.
I have read all the terms and conditions of this charter and I am committing to these terms and conditions.

[signature]	Julien SAISON	1 5 - 1 0 - 2 0 2 1
Signature	First name and last name	Date

JS.

Secretariat telephone:
....
Email:
....
Mobile phone :
....

Acceptance of the DSMB charter

Please send by mail at sa.drci.vigilance@chu-lyon.fr this form to the sponsor AND send the original documents by letter to the administrative offices of the HCL at: Siège administratif des HCL, DRCI, Vigilance des essais, 3 quai des célestins, 69002 LYON – FRANCE.

I, Fernando Santos———————————— ~~(specify your first name and last name)~~, Data Safety Monitoring Board (DSMB) member, for the FLUCOLITH study, agree with the terms of this charter V3.0 of 23/11/2020. If the charter is significantly changed, all the DSMB members will review the modifications and will give approval of the new charter.

Declaration of conflict of interest

I, Fernando Santos———————————— ~~(specify your first name and last name)~~, DSMB member as a ——— pediatric nephrologist——— ~~(specify your specialty)~~ expert for the FLUCOLITH study, confirm agreement with the following points:

Con formato: Fuente: Sin Cursiva, Color de fuente: Automático

I will undertake to :

- Protect the rights, safety and well-being of human subjects involved in the trial;
- Preserve the study integrity ;
- Be free from preconceived ideas and prejudices;
- Respect scientific and ethical standards, and eliminate or divulge real or apparent conflicts of interest during my participation to the clinical study.

Furthermore:

I declare that my spouse or my children, my employer or I have no financial interest in the study.

I undertake not to interfere with the analysis or the publication of the study results.

I undertake not to have a part-time work or a full time work, paid or unpaid in all the organizations which are : (a) involved in the study; (b) whose the products will be used or tested in the study, or whose the products or services will be directly and predictably importantly affected by the study results;

I undertake not being a leader, member, owner, fiduciary, administrator, expert or consultant of these organizations.

Confidentiality

I understand that pursuant to the Article R. 5121-13 of the French Public Health Code, I undertake not to disclose the scientific or technical confidential information which belong to the sponsor (mentioned below as "confidential information") coming to my knowledge during my DSMB participation.

I undertake, during the study and for five (5) years after the end of the study for any reason whatsoever, to:

- Protect, keep strictly confidential and process with degree of caution and protection, at least equal to the degree that I allow to my own confidential information, all the confidential information which I could know ;
- Not divulge the confidential information, directly or indirectly, to any third party ;
- Not use the confidential information, fully or partially, for a purpose other than the purpose defined in this charter without the prior and written consent of the sponsor ;
- Not request an industrial property title or exercise intellectual property rights or any other rights about confidential information communicated under this current DSMB;
- Not copy, reproduce or duplicate the confidential information, partially or completely, when such copies, reproductions or duplications were not approved by the sponsor.

Confidentiality obligations will apply only to the confidential data which will be expressly granted as confidential by the sponsor during their release in whatever form.
For this purpose, the sponsor will transmit confidential data with the mention CONFIDENTIAL on the hardware support of the divulged information.

Without hardware support, the sponsor undertakes to expressly inform you about the confidentiality nature of the information and will confirm you the confidential nature in writing as soon as possible and not later than 30 days after their release.

Any confidential data and their reproductions transmitted by the sponsor to your attention will stay the sponsor property and should be restitute immediately to the sponsor upon request.

Except as provided above, you will have no obligation and will not be subject to any restriction with regard to all confidential data which you will be able to demonstrate:

- That they have entered the public domain prior to their disclosure or after it, but in this case without any fault attributable to you; or
- That they are already known; this previous knowledge being able to be demonstrated by the existence of appropriated documents in your records; or
- That they have been sent to you by third party in a lawful manner, without restriction or violation of this charter; or
- That they have been published without contravening the terms of this charter; or
- That they have not been designated or confirmed as confidential data in the present charter.

It is expressly agreed that the release and the provision of confidential data by the sponsor under this Charter cannot be under any circumstances interpreted as conferring to you any right (under a license or by some other means) expressly or implied about topics, inventions or discoveries to which these confidential data relate.

The same is true for the authors 'rights or other rights related to the literary and artistic property, trademarks or trade secrets.

Furthermore, it is agreed that the property right on all confidential data that sponsor will disclose to you under this Charter belongs in any case to the sponsor, respecting the rights of third parties.

I will receive information from the Hospices Civils de Lyon, the study sponsor, including exclusive and confidential information.

I understand that I will have an access to records in order to participate to the study DSMB.
In my current role of DSMB member as expert, I will not publish or reproduce these documents under any circumstances.
I recognize that I do not use these documents except for my missions as a study member.

I shall take all necessary precautions to prevent any unauthorized person from having access to these records.
I shall keep all documentation confidential up to the end of the study.
I have read all the terms and conditions of this charter and I am committing to these terms and conditions.

Fernando Santos |__1|2__|-|1__|0__|-
|2__|0__|2__|1__|

Signature First name and last name Date
