# Supplementary material for: Fluconazole in hypercalciuric patients with increased 1,25(OH)2D levels: the prospective, randomized, placebo-controlled, double-blind FLUCOLITH trial
Source: Trials. 2022 Jun 16;23:499. doi: 10.1186/s13063-022-06302-z (PMC9204961; doi:10.1186/s13063-022-06302-z)
Supplement: Supplementary file 5 — Additional file 5. Flucolith_Manuel laboratoire_V5R1. [file 13063_2022_6302_MOESM5_ESM.pdf]

Manuel de laboratoire

V5 du 07 Juillet 2021

FLUCOLITH

Le fluconazole: un nouvel outil thérapeutique pour les patients hypercalciuriques avec 1,25$(OH)_2$D augmentée

**Annexes**	- Feuille de protocole infirmier « Epreuve de charge calcique »<br><br>- Planches d'étiquettes pour les analyses centralisées:<br>- Analyse génétique<br>- Vitamine D<br>- FGF23<br>- Protéine Klotho<br>- PAL totales, PAL osseuses et PTH<br>- Biocollection : plasma, sérum, urines<br><br>- Fiches de traçabilité pour les prélèvements d'analyses centralisées : V1, V2, V8, V9<br><br>- Fiche de traçabilité pour les prélèvements de la biocollection<br><br>- Bon d'accompagnements pour les analyses centralisées

HCL HOSPICES CIVILS DE LYON
CIC Centre d'Investigation Clinique LYON
Inserm La science pour la santé From science to health

Table des matières

I- Tableau des examens 3
II- Tableau des analyses biologiques sanguines 3
III- Tableau des analyses biologiques urinaires sur 24h 5
IV- Procédures des prélèvements analysés en local 6
1- Analyses en local 6
1.1 Recueil urinaire 6
1.2. Test de charge calcique 7
1.3. Test de grossesse 9
1.4. Analyses mycologiques 9
2- Critères biologiques d'arrêt de l'étude 10
V- Procédures de techniquage des prélèvements en central 11
1- Prélèvements à envoyer au Centre Hospitalier Lyon Sud 11
2- Prélèvements à envoyer au Centre Hospitalier de Caen 12
3- Prélèvements à envoyer au Groupement Hospitalier Nord (Lyon) 13
4- Prélèvements pour la biocollection 14
VI- Procédure étiquettes et traçabilité 15
VII- Procédure transporteur 15
VIII- Contacts utiles 16

I- Tableau des examens

Visites<br>Lieu	V_1<br>Hôpital consultation	V_2<br>Hôpital de jour	V_3<br>Domicile	V_4<br>Hôpital consultation	V_5<br>Home	V_6<br>Hôpital consultation	V_7<br>Hôpital consultation (<18 ans seulement)	V_8<br>Hôpital analyse	V_9<br>Hôpital de jour	V_{10}<br>Laboratoire de ville
Périodes	*Inclusion*	*Randomisation*	*Période de titration*			*Période de titration*				*Sécurité*
Semaines (± jours) / Examens	$S_{-2\pm4}$	$S_{0\pm4}$	$S_{2\pm4}$	$S_{4\pm4}$	$S_{6\pm4}$	$S_{8\pm4}$	$S_{12\pm4}$	$S_{16\pm4}$	$S_{18\pm4}$	$S_{20\pm4}$
Consentement	X									
Randomisation		X								
Examen clinique	X	X		X		X		X	X	
Dispensation du traitement		X		X		X		X		
Compliance				X		X	X	X	X	
Analyse urines de 24 heures	X		X	X	X	X		X		
Test de grossesse	X	X		X		X	X	X	X	
Analyses sanguines	X	X	X	X	X	X	X	X	X	X
Biocollection (sérum, plasma et urine)		X							X	
Test de charge calcique		X							X	
Questionnaire qualité de vie		X							X	
Questionnaire satisfaction du traitement									X	
Consultation diététique		X							X	
Electrocardiogramme	X			X		X		X		
Echographie rénale		X							X	
DXA		X								
Prélèvements mycologiques buccal et urinaire		x				x		x		
Analyse génétique (si non déjà réalisée)		x								
Effets indésirables	x	x	x	x	x	x	x	x	x	x
Traitements concomitants	x	x	x	x	x	x	x	x	x	

II- Tableau des analyses biologiques sanguines

Analyse	V1	V2	V3	V4	V5	V6	V7	V8	V9	V10
**NFS**										
Hématies	x			x		x	x	x		x
Hématocrite	x			x		x	x	x		x
Hémoglobine	x			x		x	x	x		x
Leucocytes	x			x		x	x	x		x
Plaquettes	x			x		x	x	x		x
**Ionogramme**										
Calcium	x			x		x	x	x		x
Calcium ionisé	x							x		
Phosphore	x			x		x	x	x		x
Magnésium	x							x		
**Bilan hépatique**										
ALAT	x		x	x	x	x	x	x		x
ASAT	x		x	x	x	x	x	x		x
Bilirubine	x			x		x	x	x		x
γ-GT	x			x		x	x	x		x
LDH	x			x		x	x	x		x
PAL totales	x							x		
PAL osseuses	x							x		
**Métabolisme phospho-calcique**										

Cortisol	x			x		x	x	x		x
PTH	x							x		
FGF23	x							x		
Klotho	x							x		
25-OH-D	x							x		
1,25(OH)2D	x							x		
24-25(OH)2D		x							x	
**Fonction rénale**										
Créatinine	x			x		x	x	x		x
Albumine	x			x		x	x	x		x
Protéines totales	x			x		x	x	x		x

Tableau 1: Analyses sanguines

** En bleu : analyses en centralisées*

III- Tableau des analyses biologiques urinaires sur 24h

Analyse	V1	V2	V3	V4	V5	V6	V7	V8	V9	V10
Volume	x		x	x	x	x		x		
Phosphate	x		x	x	x	x		x		
Calcium	x		x	x	x	x		x		
Sodium	x		x	x	x	x		x		
Créatinine	x		x	x	x	x		x		
Urée	x		x	x	x	x		x		
Citrate	x							x		
TmP/GFR *(calcul automatique sur eCRF)*	x							x		

Tableau 2: Analyses urinaires sur 24h

IV- Procédures des prélèvements analysés en local

1- Analyses en local

Les prélèvements analysés en local sont réalisés selon les pratiques habituelles du centre.

Le matériel utilisé est celui du centre investigateur.

1.1 Recueil urinaire

Numéro des visites concernées : V1, V3, V4, V5, V6 et V8.

Un recueil urinaire sur 24h est à réaliser par le patient à son domicile dans les 4 jours suivant la visite d'inclusion V1 et dans les 4 jours avant chacune des 5 autres visites citées ci-dessus.
Pour la visite V4, le nombre de piluliers à récupérer lors de la visite à l'hôpital sera ajusté en fonction des résultats de la calciurie. **Il est donc impératif de connaitre la calciurie le jour de cette visite.**

a. Organisation

Les cantines urinaires seront récupérées au domicile du patient par une IDE à domicile, puis déposées en laboratoire de ville pour analyse (cf. *POS Gestion IDE à domicile). Si refus des IDE, le patient déposera sa cantine lui-même au laboratoire de ville de son choix. Les laboratoires pourront être contactés en amont par les ARC des centres afin de convenir que les patients ne perdent pas de temps lors du dépôt de leur cantine.*
Pour les visites V3 et V5, les cantines seront collectées et déposées au laboratoire par les IDE, en même temps que le prélèvement sanguin pour dosage des transaminases. L'IDE est à choisir et les rendez-vous sont à prévoir par le centre investigateur, ou par le patient s'il en connait. Selon la convenance des patients, ils pourront également déposer leur cantine urinaire dans un laboratoire d'analyses médicales et réaliser le dosage des transaminases sur place le même jour.
Il est important de rappeler aux patients que le recueil des urines de 24h doit débuter la veille du dosage des transaminases.

La gestion de la collecte et du transport des cantines en laboratoire de ville, ainsi que les modalités de remboursement sont détaillées dans les procédures *Gestion IDE à domicile* destinée aux centres et *Instructions à destination de l'IDE à domicile*.

Les résultats seront envoyés par les laboratoires de ville aux coordonnées du centre indiquées sur les ordonnances. Les ARC investigation de chaque centre devront ensuite saisir ces résultats dans l'eCRF. Ils devront aussi s'assurer que les résultats soient récupérés après la visite V1 et avant les visites V2,

V3, V4, V5, V6 et V8, notamment lorsque la calciurie est nécessaire à un ajustement de la posologie (V3, V4, V5).

1.2. Test de charge calcique

Numéro des visites concernées : V2 et V9.

Une analyse des résultats de charge calcique sera effectuée en centralisée par les Dr Laurence Dubourg et Sandrine Lemoine.

Préalable et informations générales:

- Régime sans calcium les 3 jours précédents

- Vérifier que le régime ait été suivi et que le patient est à jeun d'eau et d'aliments

- Vérifier les traitements (pas de prise de diurétique)

- Pour le recueil des données source, vous pouvez vous servir du tableur Excel proposé « *Epreuve de charge calcique* » si vous le souhaitez. Vous pouvez également recueillir les données selon les procédures de votre centre.

- Après prise du calcium le patient reste à jeun, en ambulatoire dans la salle d'attente, ne doit ni manger ni boire pendant tout l'examen

- Il a pour consigne d'apporter une collation qu'il pourra prendre à la fin de l'examen

1) A l'arrivée

⇨ Le patient doit être **à jeun**

- Explication de l'examen et des consignes

- Prise des données cliniques : poids et taille

2) Temps T0

- Poser un cathlon au patient

- Prélever S0 (*cf tableau ci-dessous*)

- Faire uriner le patient et déclencher le chronomètre (*up-count*) juste après la 1ère miction (**ne pas l'arrêter avant la fin totale de l'examen**)

- Mesurer le volume avec l'éprouvette et bien le noter dans la case correspondante

- Garder les urines U0 pour les prélèvements urinaires (*cf tableau ci-dessous*)

3) Prise du calcium :

Une dose de carbonate de calcium, **correspondant à la dose journalière de calcium recommandée selon l'âge du patient**, devra être administrée :

Age des patients	Dose (mg)
<12 ans	1000
12 ans et +	1200

- Diluer la dose prescrite de calcium dans 100 ml d'eau.

ATTENTION : bien homogénéiser ; la dose de calcium doit être totalement diluée

- Faire boire le patient

- Redonner 100 mL d'eau seule dans le verre pour compléter à 200 ml

4) **Temps T1 = 2h après la prise de calcium (120 minutes)**

Respecter au maximum le délai de 2h après la prise de calcium (attention le temps total depuis la première miction sera > 2h).

- Prélever S1 sur le cathlon (*cf tableau ci-dessous*) et noter le temps exact du chronomètre

- Faire uriner le patient et noter précisément l'heure réelle inscrite sur le chronomètre et le volume de la miction

- Garder les urines U1 pour les prélèvements urinaires (*cf tableau ci-dessous*)

5) **Temps T2 = 4h après la prise de calcium (240 minutes)**

Respecter au maximum le délai de 4h après la prise de calcium (attention le temps total depuis la première miction sera > 4h).

- Prélever S2 sur le cathlon (*cf tableau ci-dessous*)

- Faire uriner le patient et noter précisément l'heure réelle inscrite sur le chronomètre et le volume de la miction

- Garder les urines U2 pour les prélèvements urinaires (*cf tableau ci-dessous*).

6) **Fin d'examen**

- Envoyer les tubes de sang S1 et S2 et les tubes d'urine U0, U1, U2.

- Le patient peut prendre la collation qu'il a amenée.

Temps (minutes)	***Prélèvements sanguins***	***Prélèvements urinaires***
**T0 (0min)**	- Na, K, Cl, bicarbonates, urée, créatinine, glucose, Ca, P, Mg, acide urique<br>- calcium ionisé<br>- PTH<br>- 25-OH-Vit D	- Bandelette urinaire<br>- Créatinine, Na, K, Cl, Ca, P, protéines

	- 1-25-OH-vit D	
**T1 (T2h=120min)**	- Ca, P<br>- calcium ionisé<br>- PTH	Créatinine, Ca, P
**T2 (T4h=240min)**	- Ca, P<br>- calcium ionisé<br>- PTH	Créatinine, Ca, P

1.3. Test de grossesse

Numéro des visites concernées : V1, V2, V4, V6, V7, V8 et V9

Un test de grossesse urinaire doit être réalisé pour chaque visite sur site, chez toutes patientes en âge de procréer, y compris les adolescentes.

Les tests sont à fournir par le centre investigateur.

1.4. Analyses mycologiques

Des prélèvements mycologiques sont réalisés aux visites V2, V6 et V8 afin d'évaluer l'apparition de résistance dans la flore buccale et urinaire. Le matériel nécessaire (écouvillons buccaux et pots pour prélèvement urinaire) est à fournir par chaque centre.

Les analyses mycologiques sont à effectuer par les laboratoires de mycologiques locaux de chaque centre investigateur.

En cas d'apparition de souches résistantes, une seconde lecture centralisée devra être effectuée par le CHU de Lyon Croix-Rousse.

La procédure relative à l'analyse et à l'envoi en centralisé est détaillée paragraphe V-3, page 13 du présent manuel.

2- Critères biologiques d'arrêt de l'étude

Ci-dessous, un rappel des critères d'arrêt (cf. protocole chapitre *7.4 Rules for a temporary or permanent discontinuation*) :

- **Augmentation des transaminases :**

→ **de 2 à 5N** : arrêt du traitement et contrôle au bout de 4-5 jours, si absence de normalisation : avis spécialisé

→ **à plus de 5N** : avis spécialisé.

- **Neutropénie** :

→ **< 1 000 G/L** : arrêt du traitement et contrôle au bout de 10 jours.

→ **< 500 G/L**: avis spécialisé.

- **Thrombopénie** :

→ **< 150 000 G/L** : arrêt du traitement et contrôle au bout de 10 jours

→ **< 80 000 G/L** : avis spécialisé.

- **Hypophosphatémie** :

→ **< 0,8 mmol/L** : contrôle à 1 semaine, en cas de persistance : arrêt du traitement

→ **< 0,5 mmol/L** : arrêt du traitement et prise en charge adaptée à la discrétion de chaque investigateur.

- **Augmentation de la créatininémie de 20% ou DFG < 60 ml/min/1,73m²** : arrêt du traitement, contrôle de la créatininémie au bout de 3-4 jours et en cas de persistance : avis spécialisé.

- **25-OH-D < 20 nmol/L** : arrêt du traitement. Mise en place d'un traitement quotidien par cholécalciférol. (gouttes) Contrôle après 1 mois.

V- Procédures de techniquage des prélèvements en central

1- Prélèvements à envoyer au Centre Hospitalier Lyon Sud

Visites concernées : V1 et V8

Analyse	Prélèvement	Coagulation	Centrifugation	Aliquotage	Stockage
**PAL totales**<br>**PAL osseuses**<br>**PTH**	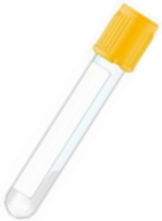<br>Tube SST<br>**Jaune d'Or**<br>de **5** mL	- Mélange par retournement x5<br><br>- Coagulation du tube pendant 30 min	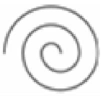<br>2000g<br>10min<br>**T° amb**	Transfert de **700 µL** min de sérum dans un cryotube étiqueté :<br>**PALt + PALos + PTH**<br>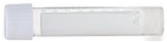	Stockage à **-80°C**
**FGF23**	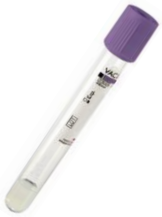<br>Tube EDTA K2<br>**Mauve**<br>de **4** mL	-	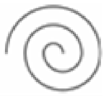<br>2000g<br>10min<br>**T° amb**	Transfert de **450 µL** min du plasma dans un cryotube étiqueté :<br>**FGF23**<br>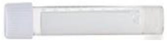	Stockage à **-80°C**
**Klotho**	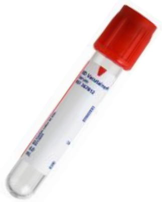<br>Tube sec<br>**Rouge**<br>de **4** mL	- Mélange par retournement x5<br><br>- Coagulation du tube pendant 30 min	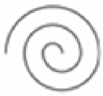<br>2000g<br>10min<br>**T° amb**	Transfert de **450 µL** min de sérum dans un cryotube étiqueté :<br>**Klotho**<br>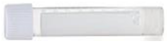	Stockage à **-80°C**

- Ne pas oublier d'étiqueter les tubes avec identifiant du patient dans l'étude, numéro et date de visite, nom de l'analyse (*cf. planche d'étiquettes fournie*).
/!\ Remettre du scotch autour du tube pour protéger l'étiquette.

- Penser à remplir la fiche de traçabilité des échantillons.

- Envoi à la fin de chaque période de suivi avec le bon d'accompagnement **(Coordonnées du médecin prescripteur et du centre pour renvoi des résultats)**: en **CARBOGLACE**

A l'attention de **C. ROGER**
Laboratoire de biochimie et biologie moléculaire
Bâtiment 3D - Hôpital Lyon Sud
165 chemin du Grand Revoyet - 69495 PIERRE BENITE

2- Prélèvements à envoyer au Centre Hospitalier de Caen

Visites concernées : V2 et V9

Analyse	Prélèvement	Coagulation	Centrifugation	Aliquotage	Stockage
**24-25(OH)$_2$D**<br>***A l'abri de la lumière***	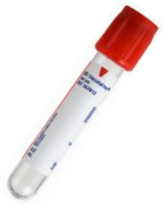<br>Tube sec<br>**Rouge**<br>de **5** mL	-	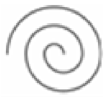<br>2000g<br>10 min<br>**T° amb**	Transfert de **500 µL** min de sérum dans un cryotube étiqueté :<br>**Vitamine D**<br>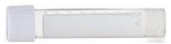	Stockage à<br>**-20°C**<br>(ou -80°C)<br>**à l'abri de la lumière**
**Analyse génétique**<br>***A V2 uniquement si non déjà réalisé***	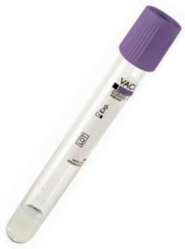<br>Tube EDTA K2<br>**Mauve**<br>de **4** mL	-	-	Transfert de **2 mL** min de sang total dans un tube étiqueté :<br>**Génétique**<br>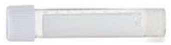	Stockage à<br>**-20°C**<br>(ou -80°C)

- Le bilan d'orientation demandé sur le bon Vit D est complété par rapport aux résultats de la V1

- Ne pas oublier d'étiqueter les tubes avec identifiant du patient dans l'étude, numéro et date de visite, nom de l'analyse (*cf. planche d'étiquettes fournie*).
/!\ Remettre du scotch autour du tube pour protéger l'étiquette.

- Penser à remplir la fiche de traçabilité des échantillons.

- Envoi à la fin de chaque période de suivi avec bon d'accompagnement + Consentement pour l'analyse génétique **(Coordonnées du médecin prescripteur et du centre pour renvoi des résultats)**:

* Analyse génétique : en **CARBOGLACE**

A l'attention de **A. MOLIN**
Service de Génétique – Pôle Biologie et Pharmacie
Centre de Référence Constitutif Rouen – Caen Maladies Rares du Métabolisme du calcium et du phosphate
CHU de Caen – Site Clémenceau
Avenue de la Côte de Nacre CS30001 – 14033 CAEN Cedex 9

* Vitamine D : en **CARBOGLACE**

Dr **NOWOCZYN** / Dr **CESBRON**
Accueil biologique commun - Laboratoire de Biochimie
CHU de Caen – Site Côte de Nacre (Niveau 3)
Avenue de la Côte de Nacre CS30001 – 14033 CAEN Cedex 9

3- Prélèvements à envoyer au Groupement Hospitalier Nord (Lyon)

Visites concernées : V2, V6 et V8 **UNIQUEMENT EN CAS DE RESISTANCES**

Les analyses mycologiques buccales et urinaires sont à réaliser en local.

Le document *Instruction à destination du Laboratoire de Mycologie* (*cf. annexe*) doit être remis à l'équipe du laboratoire de mycologie du centre investigateur, après avoir préalablement complété les coordonnées de la personne à contacter en cas de résistance détectée.

Les laboratoires locaux de mycologie réaliseront une culture de 72h sur milieu spécifique type Can2, Chromagar…

Concernant le test de sensibilité aux antifongiques Fluconazole et Voriconazole, un test type E-test sera utilisé.

A envoyer en centralisé uniquement en cas de résistances :

Les souches isolées seront conservées à **-80°C**. En effet, en cas d'apparition de souches résistantes, une **2ème lecture sera faite en centralisée**, au laboratoire de mycologie du **Groupement Hospitalier Nord de Lyon**.

- ➔ Envoi à la fin de chaque période de suivi avec le bon d'accompagnement **(Coordonnées du médecin prescripteur et du centre pour renvoi des résultats)**: en **CARBOGLACE**

> A l'attention du Dr **D. DUPONT**
> Service de mycologie
> Hôpital de la Croix-Rousse
> 103 Grande Rue de la Croix-Rousse - 69317 Cedex 04 LYON

4- Prélèvements pour la biocollection

Visites concernées : V2 et V9

Analyse	Prélèvement	Coagulation	Centrifugation	Aliquotage	Stockage
**Serum**	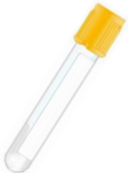 Tube SST **Jaune d'Or** de **3.5** mL	- Mélange par retournement x5<br><br>- Coagulation du tube pendant 30 min	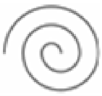 2000g<br>10 min<br>**T° amb**	Transfert de **500 µL** min de sérum dans **DEUX** cryotubes d'1,8mL étiquetés :<br>**Biocollection - sérum** 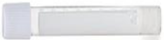	Délai de congélation = 2h<br><br>Stockage à **-80°C**
**Plasma**	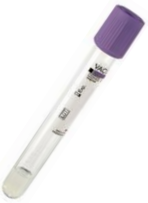 Tube EDTA K2 **Mauve** de **4** mL	-	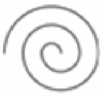 2000g<br>10 min<br>**T° amb**	Transfert de **500 µL** min de plasma dans **DEUX** cryotubes d'1,8mL étiquetés :<br>**Biocollection - plasma** 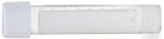	Délai de congélation = 2h<br><br>Stockage à **-80°C**
**Urines**	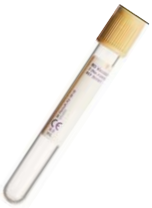 Tube Urine Chimie **Beige**	-	-	Transfert de **1mL** d'urine dans **DEUX** cryotubes d'1,8mL étiquetés :<br>**Biocollection - urines** 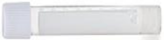	Délai de congélation = 2h<br><br>Stockage à **-80°C**

- Les cryotubes à utiliser doivent avoir un bouchon vissé comportant un joint.

- Le techniquage doit être fait le plus stérilement possible.

- Ne pas oublier d'étiqueter les tubes avec identifiant du patient dans l'étude, numéro et date de visite, nom de l'analyse (*cf. planche d'étiquettes fournie*).
/!\ Remettre du scotch autour du tube pour protéger l'étiquette.

- Penser à remplir la fiche de traçabilité des échantillons et le fichier excel.

- Les cryotubes seront rangés dans une boite, à fournir par le centre.

- Envoi à la fin de chaque période de suivi avec le bon d'accompagnement: en **CARBOGLACE**

NEUROBIOTEC
Groupement Hospitalier Est
59 Bd Pinel
69677 BRON CEDEX

VI- Procédure étiquettes et traçabilité

Des planches d'étiquettes, fiches de traçabilité et bons d'accompagnement pour les prélèvements à analyser de manière centralisée sont fournies dans le classeur investigateur.

VII- Procédure transporteur

Le transporteur intervenant dans le cadre de l'étude est BioLogistic.

L'envoi des prélèvements est à effectuer de manière groupée, à la fin de chaque période de suivi.

Les envois seront prévus et coordonnées par le centre coordinateur, en fonction des disponibilités du centre investigateur et du destinataire.

- **Le jour de l'enlèvement**

⇨ Remettre au transporteur :

- les échantillons étiquetés.
- le(s) bon(s) d'accompagnement(s) complété(s).

⇨ Le transport de ces échantillons se fera **en carboglace.**

⇨ Classer le bon d'enlèvement remis par le transporteur sur site dans le dossier de l'étude.

VIII- Contacts utiles

❖ Equipe de Coordination

- Chefs de projet : Valérie Laudy – 04 27 85 77 22 – valerie.laudy@chu-lyon.fr

Sacha Flammier – 04 72 68 13 49 – sacha.flammier@chu-lyon.fr

- ARC coordination : Camille VALLA– 04 27 85 63 17 – camille.valla@chu-lyon.fr

- Secrétariat CIC : 04 27 85 62 53

❖ Contacts biologie

- *Analyses centralisées au CHU Lyon Sud :*

  Dr Christelle Roger
  Laboratoire de Biochimie et Biologie Moléculaire - Hôpital Lyon Sud - CBAPS
  04 78 86 30 48 - christelle.roger@chu-lyon.fr

- *Relecture centralisée des analyses mycologiques au CHU Lyon Nord :*

  Dr Damien Dupont
  Institut des Agents Infectieux Parasitologie Mycologie Médicale - Centre de Biologie Lyon Nord
  04 72 00 15 20 – damien.dupont@chu-lyon.fr

- *Analyses centralisées au CHU de Caen :*

  - Vitamine D : Dr Alexandre Cesbron
  Laboratoire de Biochimie – Site Côte de Nacre – CHU de Caen
  02 31 06 50 37 ou 02 31 06 52 14 – cesbron-a@chu-caen.fr

  - Analyse génétique : Dr Arnaud Molin
  Service de Générique – Site Clémenceau - CHU de Caen
  02 31 06 45 02 ou 02 31 27 26 41 – molin-a@chu-caen.fr

- *Stockage de la biocollection*

  Nathalie Dufay
  Neurobiotec – Groupement Hospitalier Est – Hospices Civils de Lyon
  04 72 68 49 02 – nathalie.dufay@chu-lyon.fr
