# Supplementary material for: Fluconazole in hypercalciuric patients with increased 1,25(OH)2D levels: the prospective, randomized, placebo-controlled, double-blind FLUCOLITH trial
Source: Trials. 2022 Jun 16;23:499. doi: 10.1186/s13063-022-06302-z (PMC9204961; doi:10.1186/s13063-022-06302-z)
Supplement: Supplementary file 6 — Additional file 6. NIFC_Adultes_V4_20210322_FLUCOLITH smaR1. [file 13063_2022_6302_MOESM6_ESM.pdf]

Direction de la Recherche Clinique et de l'Innovation	**NOTE D'INFORMATION A L'ATTENTION DES PATIENTS**

Le fluconazole: un nouvel outil thérapeutique pour les patients hypercalciuriques avec 1,25(OH)$_2$D augmentée

Etude randomisée contre placebo

FLUCOLITH

N° EudraCT : 2020-003011-97

Version V4 du 22/03/2021

Promoteur (représenté par son représentant légal en exercice) :

Hospices Civils de Lyon
BP 2251
3 quai des Célestins,
69229 LYON cedex 02

Investigateur coordonnateur :

Dr Aurélia BERTHOLET-THOMAS
Service de Néphrologie Pédiatrique
Hôpital Femme-Mère-Enfant/Groupement Hospitalier Est
59 Bd Pinel – 69 677 BRON
Tél : 04.27.85.61.28

Madame, Monsieur,

Votre médecin ou un investigateur vous a présenté l'étude FLUCOLITH et sollicite votre accord pour que vous y participiez. Ce document a pour objectif de vous donner toutes les informations relatives à cette étude de façon à vous permettre d'exercer au mieux votre liberté de décision. Ce document est obligatoire et son contenu est défini par le ***Code de la Santé Publique, article L 1122-1*** *régissant les recherches impliquant la personne humaine.*
Il décrit précisément l'étude et mentionne toutes les autorisations réglementaires obtenues pour sa mise en œuvre.
Avant de prendre une décision, il est important que vous lisiez attentivement ces pages qui vous apporteront les informations nécessaires concernant les différents aspects de cette étude. Vous devez conserver ce document. N'hésitez pas à poser des questions si vous ne comprenez pas certains éléments.
La signature du formulaire de consentement devenue obligatoire par l'application du code de la Santé Publique (livre I, titres 2 et 3 du CSP), n'affecte aucunement vos droits légaux.
Votre participation est entièrement volontaire. Si vous ne désirez pas prendre part à cette étude, vous continuerez à bénéficier de la meilleure prise en charge médicale possible, conformément aux connaissances actuelles.

Pourquoi cette recherche?

Vous avez présenté ou vous présentez des lithiases urinaires éventuellement associées à une néphrocalcinose.
Votre médecin a mis en évidence une hypercalciurie (présence anormalement élevée de calcium dans les urines) en lien avec une augmentation de la forme active de la vitamine D (1,25$(OH)_2$D). Cela a pour conséquence d'augmenter l'absorption du calcium par les intestins dont l'excès est ensuite éliminé par les reins.
Les lithiases et/ou la néphrocalcinose sont directement secondaires à cette hypercalciurie. Elles peuvent à terme conduire à une dégradation de la fonction rénale (insuffisance rénale à des stades différents) et à des complications osseuses (douleurs, fractures, ostéoporose, rachitisme).
Ces anomalies biologiques peuvent dans certains cas être d'origine génétique. Ces anomalies génétiques sont actuellement recherchées en pratique courante. Si vous n'en avez pas encore bénéficié, un test génétique sera pris en charge dans le cadre du protocole. La détermination de votre profil génétique pourra permettre de vous classer en répondeur/non-répondeur au traitement par fluconazole en fonction de l'anomalie génétique identifiée.

Le contrôle de la calciurie est un critère important dans votre prise en charge. En effet, la normalisation ou la diminution de la calciurie peut prévenir ou ralentir la progression des néphrolithiases/néphrocalcinoses, et donc les complications à long terme.

Il n'existe à l'heure actuelle aucun traitement spécifique en dehors des mesures hygiéno-diététiques (hyperhydratation, alcalinisants, limitation des apports sodés), et dans certains cas un traitement par hydrochlorothiazide.

Le fluconazole est un médicament antifongique utilisé de longue date chez l'adulte et l'enfant contre les infections mycosiques (champignons). Le métabolisme du fluconazole est bien connu, même chez l'enfant, et sa toxicité est faible. Il est également connu pour diminuer les taux de 1,25$(OH)_2$D, par inhibition de l'enzyme qui la produit. En effet, de récentes études ont permis de prouver que le fluconazole diminue la calciurie chez quelques patients avec des taux d'1,25$(OH)_2$D augmentés.

FLUCOLITH est la première étude de grande envergure dont l'objectif est de démontrer l'efficacité du fluconazole dans la diminution la calciurie chez l'enfant et l'adulte hypercalciuriques avec 1,25$(OH)_2$D augmentée.
Pour prouver son efficacité, le fluconazole sera comparé à un placebo : les patients inclus dans l'essai seront tirés au sort (randomisation) et seront répartis dans deux groupes de traitement : un groupe recevra le fluconazole, et l'autre un placebo. Ni le médecin ni le patient ne sauront quel est le traitement reçu par le patient (double -aveugle).
Votre traitement habituel ne sera pas modifié (en dehors de certains traitements médicamenteux dont l'hydrochlorothiazide) ; il n'est donc pas attendu d'aggravation de la maladie en cours de l'étude.
L'étude se concentrant sur l'efficacité du fluconazole sur la calciurie, il n'est pas attendu de bénéfices directs sur la survenue de coliques néphrétiques. L'efficacité du fluconazole sur les coliques néphrétiques ainsi que sur la néphrocalcinose nécessitera la réalisation d'une autre étude avec un suivi plus long.

Quel est l'objectif de cette recherche?

L'objectif principal de l'étude *FLUCOLITH* est de démontrer que le fluconazole normalise ou fait diminuer significativement le taux de calcium urinaire après 4 mois de traitement chez les patients avec hypercalciurie et 1,25$(OH)_2$D augmentée.
Nous cherchons aussi à étudier l'effet du fluconazole sur le métabolisme phospho-calcique, et à évaluer la sécurité de ce traitement.

Quelle est la méthodologie de cette recherche ?

Il s'agit d'une étude multicentrique nationale, randomisée, contrôlée en double-aveugle contre placebo, à deux groupes parallèles.
Ce projet est subventionné par le PHRC National 2019 (Programme Hospitalier de Recherche Clinique).

Votre durée de participation sera de 5 mois ½ maximum (22 semaines ± 1 semaine).

60 patients seront recrutés dans cette étude : 30 dans le groupe fluconazole et 30 dans le groupe placebo.
Les patients qui seront inclus dans cette recherche seront âgés de 10 à 60 ans, devront présenter dans leur histoire médicale une néphrolithiase et/ou une néphrocalcinose, un taux de calcium urinaire sur les urines de 24h > 0.1mmol/kg/jour, un taux de 25-OH-D sanguin ≥ 20 nmol/L, un taux de 1,25(OH)$_2$D sanguin ≥ 150pmol/L, et un taux de calcium sanguin ≤ 2.65 mmol/L.

<u>Comment va se dérouler cette recherche ?</u>

Vous serez inclus dans l'étude si votre calciurie sur 24h est > à 0.1 mmol/kg/jour, si votre taux de 25-OH-D sanguin est ≥ 20 nmol/L, si votre taux sanguin de 1,25(OH)$_2$D est ≥ à 150 pmol/L, et si votre taux de calcium sanguin est ≤ 2.65 mmol/L. Si ce n'est pas le cas, vous ne participerez pas à l'essai.
Vous aurez une chance sur deux de recevoir le fluconazole, et une chance sur deux de recevoir le placebo.

Si vous êtes inclus dans l'étude, vous aurez à réaliser 9 visites spécifiquement pour la recherche:

- 1 première visite d'inclusion à l'hôpital, en consultation (V_1),
- 5 visites à l'hôpital, dont la première et la dernière en hôpital de jour, et les autres en consultation (V_2, V_4, V_6, V_8 et V_9)
- 2 recueils d'urines sur 24 heures seront à effectuer à votre domicile (V_3 et V_5), et seront récupérés par un transporteur pour être analysés à l'hôpital.
- 1 prise de sang sera à réaliser en fin d'étude, dans le laboratoire de votre choix (V_{10}).

Voici le schéma récapitulatif des visites :

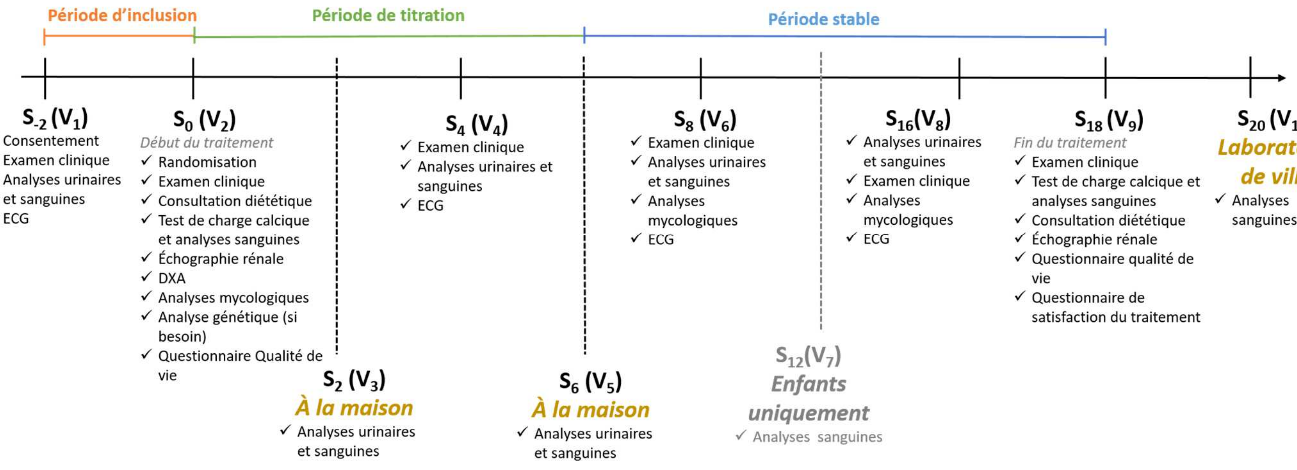

L'étude comprend deux périodes : une période de titration (6 semaines : de S_0 (V_2) à S_6 (V_5)), et une période stable (12 semaines : de S_6 (V_5) à S_{18} (V_9)).

Vous recevrez initialement une gélule de fluconazole 50mg ou une gélule de placebo par jour. Pendant la période de titration, une analyse des urines sur 24h sera effectuée toutes les 2 semaines pour adapter le nombre de gélules que vous devrez prendre :

- si votre calciurie sur 24h est > 0.1mmol/kg/jour, alors vous devrez prendre une gélule en plus par jour, jusqu'à atteindre un maximum de 4 gélules par jour à la fin de la période de titration ;
- si votre calciurie sur 24h est ≤ à 0.1mmol/kg/jour, alors vous continuerez à prendre le même nombre de gélules par jour.

***S** = semaine*
***V** = visite*

[illegible]eront à prendre tous les matins, pendant ou en dehors du petit-déjeuner.

Pour les recueils urinaires effectués à votre domicile, vous serez contacté par l'équipe investigatrice de l'hôpital pour vous informer de la posologie à adopter.
La dose déterminée à l'issue de la période de titration sera à maintenir durant la période stable.

Le tableau suivant synthétise les examens qui seront réalisés lors de chaque visite :

**Visites (V)**	**V_1**	**V_2**	**V_3**	**V_4**	**V_5**	**V_6**	**(V_7)**	**V_8**	**V_9**	**V_{10}**
Semaines	*S_{-2}*	*S_0*	*S_2*	*S_4*	*S_6*	*S_8*	*(S_{12})*	*S_{16}*	*S_{18}*	*S_{20}*
Durée des visites	*2h*	*5h*	*NA*	*1h*	*NA*	*1h*	*30mn*	*1h*	*5h*	*NA*
Présentation de l'étude et signature du consentement	X									
Mesure du poids, de la taille et des signes vitaux (pouls et tension artérielle)	X	X		X		X		X	X	
si vous avez moins de 20 ans : Evaluation du statut pubertaire (stades de Tanner)		X							X	
Recueil d'urines sur 24h	X		X	X	X	X		X		
Prise de sang[1]	X	X	X	X	X	X	X	X	X	X
Biocollection		X							X	
Test de charge calcique[2]		X							X	
Consultation diététique[3]		X							X	
Questionnaire de qualité de vie		X							X	
Questionnaire de satisfaction du traitement									X	
Echographie rénale		X							X	
Quantification de la densité minérale osseuse (DXA) [4]		X								
Analyses mycologiques (urines et bouche)		X				X		X		
Electrocardiogramme (ECG)	X			X		X		X		
si vous êtes une femme : Test de grossesse urinaire	X	X		X		X	X	X	X	
Test génétique (si non-réalisé auparavant) [5]		X								
Revue des effets secondaires	X	X	X	X	X	X	X	X	X	X
Revue des traitements concomitants	X	X	X	X	X	X	X	X	X	
Distribution / Revue du carnet patient		X		X		X	X	X	X	

Toutes les visites et examens réalisés entrent dans le cadre de la recherche.

[1] *Prise de sang* :
Analyse du métabolisme phosphocalcique et des biomarqueurs osseux, et analyses de sécurité :
- *dosages effectués localement*: fonctions hépatiques, cortisol, globules rouges, albumine, créatinine, calcium, protéines totales, calcium ionisé*, phosphore, lactate deshydrogenase, magnésium*, 25-OH-D* et 1,25$(OH)_2D$*
- *dosages centralisés à Lyon*: PTH*, phosphatases alcaline *, FGF23*, Klotho*

- *dosage centralisé à Caen* : 24-25 $(OH)_2D$**

** seulement à V_1 et V_8*
*** seulement à V_2 et V_9*
V_3 et V_5 : dosage des fonctions hépatiques uniquement

[2] *Test de charge calcique* :
Pour ce test, vous devrez être à jeun, et le rester tout au long de la procédure (durée : 4h). Vous ne devrez pas ingérer de produits laitiers 3 jours avant le test, de manière à ne pas interférer avec son interprétation.

Le test de charge calcique consiste à vous faire ingérer une dose de carbonate de calcium (correspondant à la dose journalière de calcium recommandée) ; un échantillon de sang et d'urines sera collecté avant la prise, puis 2h et 4h après la prise. Vous ne serez piqué qu'une seule fois, à l'aide d'un cathéter.
Ce test permet de mieux caractériser votre hypercalciurie et aide le médecin à adapter la prise en charge symptomatique; l'objectif est d'évaluer les caractéristiques de l'hypercalciurie sous traitement.
Les échantillons seront analysés localement, et l'interprétation du test sera centralisée à Lyon.
Pendant les 4 heures du test, vous effectuerez également les autres examens du protocole (à savoir : examen clinique avec le médecin, remplissage du/des questionnaire(s), revue des éventuels effets secondaires et traitements concomitants, remise du carnet patient). L'échographie rénale (et la DXA pour la visite V_2) seront réalisées avant ou après ces examens.

[3] *Consultation avec une diététicienne* :
- Evaluation et standardisation de votre apport calcique, sodique et protéique.
- Remplissage de 3 auto-questionnaires: évaluation de l'apport en vitamine D, en calcium (questionnaire Fardellone), et questionnaire d'exposition au soleil.

La durée de la consultation sera d'environ 30 min.

[4] *DXA (absorptiométrie biphotonique)* :
Il s'agit d'un examen d'imagerie permettant de mesurer la masse osseuse. Il est totalement indolore, rapide (3 minutes en position allongée sans bouger) et faiblement irradiant. Les images de l'examen seront centralisées au sein de l'unité 1033 « Physiopathologie, Diagnostic et traitements des Maladies Osseuses » à l'hôpital Edouard Herriot de Lyon.

[5] *Test génétique* :
Un test génétique vous sera proposé à la visite V_2 si vous n'en avez pas réalisé avant. La réalisation de ce test n'entraînera pas de piqure supplémentaire pour vous. Le but est de rechercher si votre hypercalciurie peut être expliquée par des mutations dans les gènes impliqués dans le métabolisme phosphocalcique et dans le métabolisme de la vitamine D.
Un formulaire de consentement concernant spécifiquement la réalisation de ce test vous sera remis en même temps que ce présent consentement. Vous avez le droit de refuser de réaliser ce test tout en continuant la recherche.
L'analyse de votre prélèvement génétique sera centralisée au CHU de Caen ; le prélèvement y sera conservé pendant une durée de 30 ans, sauf mention contraire de votre part sur le formulaire de consentement génétique.

Le volume de sang qui vous sera prélevé sur le total de toutes les visites sera d'environ 127.5ml (détail du volume de sang prélevé lors de chaque visite : V_1 = 24 ml ; V_2 et V_9 = 21.5 ml ; V_3 et V_5 = 3 ml ; V_8 = 24.5 ml ; V_4, V_6 et V_{10} = 10 ml).
Ces volumes restent inférieurs aux seuils définis par les recommandations nationales.

Les prélèvements de sang et d'urines effectués lors de la visite V_2 et lors de la visite V_9 constitueront une sérothèque, une plasmathèque et une urinothèque. Ils seront conservés à la fin de l'étude pour une utilisation ultérieure à des fins d'autres recherches, sauf opposition de votre part. Pour chacune de ces deux visites, 1ml de sérum, 1ml de plasma et 2ml d'urines seront conservés.
Cette collection sera réalisée conformément à la réglementation. Aucun examen génétique ne pourra se faire sans votre accord écrit.
Cette collection, anonymisée, sera stockée au sein de l'hôpital où vous aurez été inclus, et ce jusqu'à la fin de l'essai. Elle sera ensuite conservée à -80°C de manière centralisée dans une biobanque labellisée (Neurobiotec, CRB, HCL, Lyon). Les échantillons seront détruits au-delà de 5 ans suivant la fin de la recherche.

Vous pouvez refuser de participer à cette collection (même si vous participez à l'étude), et demander la destruction de vos échantillons à tout moment.
Si vous ne souhaitiez plus participer ou si vous deviez sortir de la recherche, vos échantillons resteront conservés dans la biobanque, sauf demande contraire de votre part.

Quels sont les bénéfices, les risques et les contraintes liés à votre participation ?

En participant à cette étude, vous bénéficierez d'une expertise médicale supplémentaire, l'étude étant menée dans des Centres de Référence ou des Centres de Compétences experts dans la pathologie. Vous bénéficierez notamment d'une évaluation de votre statut osseux via de nouvelles techniques d'imagerie osseuse (DXA). Aucun autre bénéfice direct n'est certain pour vous.

Afin d'éviter le biais dû à la synthèse de vitamine D induite par le soleil, il vous sera demandé de ne pas voyager dans un pays chaud pendant la durée de l'étude. De même, à partir du mois d'Avril, vous devrez appliquer les jours de fort ensoleillement de la crème solaire indice 50 sur les zones exposées avant de sortir dehors.

L'échographie rénale, la DXA et l'ECG sont indolores, ainsi que l'examen urinaire. La prise de sang peut être douloureuse et causer des bleus : il pourra vous être proposé de bénéficier d'une anesthésie locale cutanée par patch ou crème de type EMLA® si vous le souhaitez.

Le fluconazole est supposé faire diminuer la calciurie par la diminution des concentrations en $1,25(OH)_2D$. Chez cette population de patients, le risque de déficit en $1,25(OH)_2D$ induite par le fluconazole est très bas.

De manière à surveiller l'apparition de potentielles résistances mycologiques au fluconazole, une analyse de la flore des urines et de la bouche (écouvillon buccal) sera effectuée aux visites V_2, V_6 et V_8; cet examen n'est pas douloureux.

Si vous êtes une femme, vous devez vous engager à avoir une méthode de contraception hautement efficace pendant toute la durée de l'étude.
De même, si vous êtes un homme, votre partenaire devra utiliser une méthode de contraception hautement efficace, et vous devrez utiliser un préservatif.

Les effets indésirables les plus fréquemment rapportés (≥1/100 à <1/10) lors de l'utilisation du fluconazole dans son indication antifongique et classiquement réversibles à l'arrêt du traitement sont : maux de tête, douleurs abdominales, diarrhées, nausées, vomissements, augmentation des enzymes du foie, et éruption cutanée.
A noter que les doses maximales de fluconazole utilisées pour contrôler l'hypercalciurie sont moins importantes que celles utilisées dans son indication antifongique.

Si vous présentez un évènement indésirable ou un néphrolithiase au cours de votre participation à l'étude, vous serez pris en charge par une équipe médicale selon les pratiques habituelles. Votre prise en charge sera adaptée à la pathologie présentée et en accord avec les recommandations nationales.

En conclusion, il n'y a pas de risques majeurs liés à votre participation à cette étude.

<u>Quelles sont les éventuelles alternatives médicales?</u>

Pour le moment, il n'y a pas d'alternative thérapeutique ayant démontré son efficacité dans cette indication.

<u>Quels sont les traitements autorisés et non autorisés durant la recherche ?</u>

- Si vous êtes traité par hydrochlorothiazide ou par tout autre diurétique, vous devrez stopper ce traitement 2 semaines avant de pouvoir être inclus dans l'étude (soit 2 semaines avant la visite V_1). Il n'est pas attendu de conséquences cliniques pour vous à l'arrêt de ce traitement.
- De même, les traitements utilisés dans le cadre de votre pathologie devront être arrêtés pour éviter de potentielles interactions avec le fluconazole (notamment alcalinisants urinaires (citrate et bicarbonate)).
- Si vous receviez déjà du fluconazole ou du ketoconazole dans les 6 mois précédant l'inclusion, vous ne pourrez pas être recruté dans l'étude.
- Le fluconazole peut modifier le métabolisme de certains médicaments très spécifiques ; les traitements suivants sont interdits pendant la durée du traitement, et jusqu'à la fin de votre participation à l'étude:
    - Médicaments connus pour allonger l'intervalle QTc et métabolisés par le cytochrome P450 (CYP) 3A4: **érythromycine**, pimozide (neuroleptique), anti-paludéens de synthèse (quinidine et halofantrine), amiodarone (anti-arythmique).

- Médicaments pouvant perturber les concentrations d'électrolytes : diurétiques de l'anse, diurétiques thiazidiques, laxatifs et lavements, amphotéricine B, corticostéroïdes à forte dose.

Quels sont vos droits ?

- ***Participation libre et volontaire***

Vous êtes entièrement libre d'accepter ou de refuser de participer à cette recherche sans que cela ne modifie la qualité des soins auxquels vous avez droit, ou les relations existant avec votre médecin ou l'investigateur.
Si vous décidez de participer à cette recherche, mais que vous changiez d'avis au cours de celle-ci, vous pouvez à tout moment demander d'interrompre votre participation à l'étude sans aucun préjudice, sans justification de votre part et sans que votre responsabilité ne soit engagée. Dans ce cas, vos données recueillies jusque-là seront utilisées dans les résultats de l'étude.
D'autre part, s'il le juge nécessaire pour votre bien, l'investigateur pourra modifier votre suivi et vous pourrez continuer à bénéficier pleinement de sa compétence.

Plus généralement, votre participation à cette étude ne décharge en aucune façon le représentant du promoteur ou les investigateurs de leurs devoirs envers vous.

A tout moment, toutes les informations que vous souhaiteriez obtenir ultérieurement concernant cette recherche vous seront communiquées dans la mesure du possible par votre médecin et/ou par l'investigateur. Vous serez tenus informés de toute nouvelle donnée importante concernant l'étude à laquelle vous acceptez de participer.
L'investigateur, tout comme le promoteur peut interrompre à tout moment votre participation à l'étude s'il juge que cela est dans votre intérêt, ou arrêter l'étude dans sa globalité pour des raisons médicales, administratives ou autres.
Pour pouvoir participer à cette étude, vous devez nécessairement être affilié à un régime d'assurance maladie telle que celui de la sécurité sociale.

Durant toute votre participation à cette recherche et jusqu'à votre dernière visite dans l'étude (dernière prise de sang à V_{10}), il vous sera demandé de ne pas accepter de participer à une autre étude qui pourrait interférer avec les résultats du présent protocole de recherche.

Tout frais additionnel dans le cadre de la recherche sera remboursé sur présentation de justificatif. De même, tous les frais médicaux liés à l'étude seront à la charge du promoteur. Il n'y aura pas de coût supplémentaire pour vous.

- ***Confidentialités accès et protection des données***

Dans le cadre de la recherche interventionnelle à laquelle les Hospices Civils de Lyon vous proposent de participer, un traitement informatique de vos données personnelles va être mis en œuvre pour permettre d'analyser les résultats de la recherche au regard de l'objectif de cette dernière. Le responsable du traitement des données est le promoteur, dont les coordonnées figurent sur la première page de ce document. Ce traitement des données a pour fondement juridique l'article 6 du Règlement Général sur la Protection des Données (RGPD) à savoir l'exécution d'une mission d'intérêt public dont est investi le responsable de traitement et les intérêts légitimes poursuivis par lui. De plus, au titre de l'article 9 du RGPD le responsable de traitement peut de manière exceptionnelle traiter des catégories particulières de données, incluant des données de santé notamment à des fins de recherche scientifique. Pour l'analyse, vos données médicales seront transmises aux Hospices Civils de Lyon ou aux personnes ou sociétés agissant pour son compte, en France ou à l'étranger. En cas de transfert de données à caractère personnel hors de l'Union Européenne et / ou vers un pays ne garantissant pas un niveau de protection suffisant par rapport à l'Union Européenne ou à une organisation internationale, le promoteur et/ou le responsable de traitement mettront en place des garanties appropriées pour ce transfert (Clauses Contractuelles Spécifiques). Si vous souhaitez obtenir une copie des Clauses Contractuelles Spécifiques, vous pouvez vous adresser au Délégué à la Protection des Données (DPO) du promoteur à l'adresse suivante : dpo@chu-lyon.fr. Ces données seront identifiées par un code et/ou vos initiales. Ces données pourront également, dans des conditions assurant leur confidentialité, être transmises aux autorités de santé françaises ou étrangères et à d'autres entités en dehors des Hospices Civils de Lyon.
Les seules personnes autorisées à consulter votre dossier médical sous sa forme nominative (c'est-à-dire directement identifiable) sont :

- votre médecin/le professionnel de santé qui vous suit et l'équipe soignante

- le personnel dédié aux recherches au sein de l'hôpital (appelées "Attachés Recherche Clinique ARC ou Techniciens d'Études Cliniques" TEC) pour saisir les données liées à la recherche ou assurer le contrôle des données
- le personnel du promoteur intervenant pour l'assurance qualité des données (appelé Attaché de Recherche Clinique) ou les autorités de santé

Sachez que toutes ces personnes sont soumises au secret professionnel.

Les données seront transférées et collectées conformément à la méthodologie de référence MR001 de la Commission Nationale de l'Informatique et des Libertés (CNIL) pour laquelle les Hospices Civils de Lyon ont signé un engagement de conformité.
Si vous avez des questions ou des réclamations au sujet du traitement de vos données au cours de cette étude, vous pouvez contacter le DPO par voie électronique : dpo@chu-lyon.fr ou par courrier postal :
Le délégué à la protection des données
162 avenue Lacassagne
Bâtiment A – 3e étage – Bureau 316
69003 LYON
Si vous estimez, après avoir contacté le DPO des HCL, que vos droits sur vos données ne sont pas respectés, vous pouvez adresser une réclamation (plainte) à la CNIL :
https://www.cnil.fr/fr/webform/adresser-une-plainte

Si vous êtes d'accord, votre médecin traitant pourra être informé de votre participation à l'étude.

○ ***Exercer vos droits***

Vous pourrez également, à tout moment, exercer votre droit d'accès, de vérification, de correction, de limitation, d'effacement et d'opposition à la transmission des données vous concernant en en faisant la demande auprès du médecin de votre choix ou auprès d'un investigateur de l'étude. Si vous souhaitez exercer votre droit à l'effacement de vos données, le responsable de traitement peut au titre des Articles 17.3.c et 17.3.d. du RGPD ne pas faire droit à cette demande si celle-ci est susceptible de rendre impossible ou de compromettre gravement la réalisation des objectifs de la recherche. Ainsi, vos données recueillies préalablement au retrait de votre consentement pourront ne pas être effacées et pourront continuer à être traitées dans les conditions prévues par la recherche.
Si les résultats de cette étude devaient être présentés dans des communications et/ou des publications scientifiques médicales, l'identité des participants n'apparaîtra d'aucune façon.
Les données relatives à des effets indésirables qui seraient détectés dans le cadre de la présente étude doivent être collectées et traitées pour répondre à une obligation légale de vigilance et ne pourront donc donner lieu à une opposition de votre part.
A l'issue de l'étude, les résultats globaux de la recherche et votre groupe de traitement pourront vous être communiqués sur simple demande auprès de l'investigateur coordonnateur de l'étude, le Dr Aurélia BERTHOLET-THOMAS. Les données collectées dans le cadre de cette recherche peuvent être utilisées lors de nouvelles recherches conduites ultérieurement à des fins scientifiques. Vous pouvez décider de vous opposer à cette utilisation à tout moment auprès de votre investigateur.
Le Laboratoire Arrow, qui fournit gracieusement les gélules de fluconazole dans le cadre de l'essai, aura accès aux données anonymisées de Pharmacovigilance (évènements indésirables qui surviendraient en cours d'étude), ainsi qu'au rapport final des résultats à l'issue de la fin de la recherche. .

○ ***Dispositions réglementaires***

L'étude sera conduite conformément aux lignes directives des Bonnes Pratiques Cliniques françaises et européennes, à la déclaration d'Helsinki dans sa dernière version, aux recommandations de l'ICH (International Conference on Harmonisation), Guideline for Good Clinical Practice ainsi qu'aux dispositions législatives et réglementaires en vigueur.
Le Comité de Protection des Personnes Nord Ouest I a émis un avis favorable à la réalisation de cette étude le 14/12/2020. L'Agence nationale de sécurité du médicament et des produits de santé (ANSM) a également donné son autorisation à la mise en œuvre de l'étude le 24/11/2020. Enfin, cette recherche respecte le règlement général sur la protection des données.
Le promoteur de cette recherche, les Hospices Civils de Lyon, BP 2251, quai des célestins, 69229 Lyon cedex 02, a souscrit une assurance de responsabilité civile auprès de la Société Hospitalière d'Assurance Mutuelle, 18 rue Edouard Rochet, 69008 Lyon, sous le numéro 159077.

Les personnes ayant subi un préjudice après participation à une recherche interventionnelle peuvent faire valoir leurs droits auprès de l'assureur du promoteur.

L'investigateur doit vous fournir toutes les explications nécessaires concernant cette recherche. Si vous souhaitez vous en retirer à quelque moment que ce soit, et quel que soit le motif, vous continuerez à bénéficier du suivi médical et cela n'affectera en rien votre surveillance future.

Qui pouvez-vous contacter pour toute question ?

Si vous avez des questions concernant l'étude, n'hésitez pas à nous les poser. Nous pouvons vous donner les informations complémentaires que vous souhaitez. Les noms et numéros de téléphone des personnes à contacter sont les suivants :

Investigateur coordonnateur de l'étude

Dr Aurélia BERTHOLET-THOMAS
Service de Néphrologie Pédiatrique
Hôpital Femme-Mère-Enfant/Groupement Hospitalier Est
59 Bd Pinel – 69 677 BRON
Tél : 04.27.85.61.28

Investigateur de votre centre référent pour l'étude

Titre Prénom NOM : ……
Service : ………
Hôpital/Groupement : ……
Adresse :
………
………
Tél : ………………………………………………………………………

Nous vous remercions de l'attention que vous avez portée à la lecture de cette notice. Une copie de ce document vous sera remise pour que vous puissiez bénéficier de l'ensemble des informations concernant votre participation à l'étude.

Lorsque vous aurez lu cette note d'information, il vous sera proposé, si vous êtes d'accord, de donner votre consentement écrit en signant le formulaire préparé à cet effet.

HCL HOSPICES CIVILS DE LYON<br>Direction de la Recherche Clinique et de l'Innovation	**FORMULAIRE DE CONSENTEMENT A L'ATTENTION DES PATIENTS**

La loi 2012-300 du 5 mars 2012 relative aux recherches impliquant la personne humaine rend obligatoire le recueil de l'accord écrit des patients sollicités pour participer à toute recherche interventionnelle. C'est un tel accord qui vous est demandé ci-dessous, pour participer à l'étude intitulée :

Le fluconazole: un nouvel outil thérapeutique pour les patients hypercalciuriques avec 1,25(OH)$_2$D augmentée
FLUCOLITH

Version V4 du 22/03/2021

Promoteur : Hospices Civils de Lyon
BP 2251
3 quai des Célestins,
69229 LYON cedex 02

Investigateur coordonnateur :
Dr Aurélia BERTHOLET-THOMAS
Service de Néphrologie Pédiatrique
Hôpital Femme-Mère-Enfant/Groupement Hospitalier Est
59 Bd Pinel – 69 677 BRON
Tél : 04.27.85.61.28

Je soussigné(e) .... *(nom, prénom)* certifie avoir lu et compris la note d'information qui m'a été remise.

J'ai eu la possibilité de poser toutes les questions que je souhaitais au *Pr/Dr/Mr/Mme* .... *(nom, prénom)* qui m'a expliqué la nature, les objectifs, les risques potentiels et les contraintes liées à ma participation à cette recherche.

Je connais la possibilité qui m'est réservée d'interrompre ma participation à cette recherche à tout moment sans avoir à justifier ma décision et je ferai mon possible pour en informer l'investigateur qui me suit dans la recherche. Cela ne remettra naturellement pas en cause la qualité des soins ultérieurs.

J'ai eu l'assurance que les décisions qui s'imposent pour ma santé seront prises à tout moment, conformément à l'état actuel des connaissances médicales.

J'ai bien compris que l'investigateur peut interrompre à tout moment ma participation à l'essai s'il le juge nécessaire.

Je suis informé(e) de la possibilité que mes données recueillies dans le cadre de cette étude puissent être réutilisées lors de recherches ultérieures exclusivement à des fins scientifiques et que je peux m'y opposer.

Je suis informé(e) de la possibilité qu'une partie des prélèvements effectués à l'occasion de ce protocole de recherche soit conservée pour une utilisation ultérieure à des fins de recherche. J'ai également été informé(e) de mon droit à m'opposer à ce que cette conservation et cette utilisation ultérieure à des fins de recherche aient lieu.

J'ai bien noté / été informé que cette recherche a reçu l'avis favorable du Comité de Protection des Personnes Nrd Ouest le 14/12/2020 et l'autorisation de l'ANSM le 24/11/2020 et a fait l'objet d'une déclaration à la Commission Nationale Informatique et Libertés (CNIL).

J'ai bien noté que cette recherche est menée conformément aux articles L1121-1 et suivants du Code de la Santé Publique, relatifs à la protection des personnes qui se prêtent à des recherches impliquant la personne humaine et conformément à la règlementation en vigueur.

Je certifie sur l'honneur être affilié à un régime de sécurité sociale ou bénéficiaire d'un tel régime.

Le promoteur de la recherche, les Hospices civils de Lyon, BP 2251, quai des célestins, 69229 Lyon cedex 02 a souscrit une assurance de responsabilité civile en cas de préjudice auprès de de la Société Hospitalière d'Assurance Mutuelle, 18 rue Edouard Rochet, 69008 Lyon, sous le numéro 159077.

J'accepte que les personnes qui collaborent à cette recherche ou qui sont mandatées par le promoteur, ainsi qu'éventuellement le représentant des Autorités de Santé, aient accès à l'information contenue dans mon dossier médical dans le respect le plus strict de la confidentialité.

J'accepte que les données enregistrées à l'occasion de cette recherche, comportant des données génétiques puissent faire l'objet d'un traitement informatisé sous la responsabilité du promoteur.

J'ai bien noté que, conformément aux dispositions de la loi relative à l'informatique, aux fichiers et aux libertés, je dispose d'un droit d'accès , de rectification, de vérification, de correction, de limitation, d'effacement et d'opposition à la transmission de mes données couvertes par le secret professionnel susceptibles d'être utilisées dans le cadre de cette recherche et d'être traitées. Ces droits s'exercent auprès de l'investigateur qui me suit dans le cadre de cette recherche et qui connaît mon identité.

J'ai été avisé qu'aucune indemnité n'est prévue pour ma participation à cette recherche**.**

Mon consentement ne décharge en rien l'investigateur et le promoteur de la recherche de leurs responsabilités à mon égard. Je conserve tous les droits garantis par la loi.

Les résultats globaux de la recherche me seront communiqués directement, si j'en fais la demande, conformément à la loi du 4 mars 2002 relative aux droits des malades et à la qualité du système de santé.
Je peux à tout moment demander des informations complémentaires au Pr/Dr à Mr/Mme ………………………………………….

Trois exemplaires originaux de ce formulaire de consentement ont été établis : un m'a été remis, l'autre a été remis à l'investigateur et le dernier sera conservé sous enveloppe scellée par le promoteur au minimum 25 ans après la fin de la recherche.

- **<u>Patient donnant son consentement</u> :**

Ayant disposé d'un temps de réflexion suffisant avant de prendre ma décision, j'accepte librement et volontairement :

- **de participer à la recherche *FLUCOLITH***

- **que soit constituée une collection de mes échantillons biologiques qui sera conservée à l'issue de l'étude pour une utilisation ultérieure à des fins d'autres recherches :**

☐ **oui** ☐ **non**

NOM, Prénom du patient participant à la recherche :
………

Fait à : ………………………………………………………, le |___|___| / |___|___| / |___|___|___|___|

Signature du patient :

- **<u>Investigateur obtenant le consentement</u> :**

J'atteste que toutes les obligations liées à un consentement éclairé ont été satisfaites dans le cadre de ce projet de recherche clinique – que le participant a reçu une information relative à ses droits, que nous avons discuté de ce projet et que je lui ai expliqué en termes compréhensibles l'ensemble des informations contenues dans la notice. Je certifie également avoir laissé le participant me poser toutes les questions qu'il souhaitait et y avoir répondu.

NOM, Prénom de l'investigateur : ………

Fait à : ………………………………………………………, le |___|___| / |___|___| / |___|___|___|___|

Signature de l'investigateur :
